# Sulfate‐Directed Silver Dendrites with Enhanced Stability for Ultrasensitive SERS‐Based Therapeutic Drug Monitoring

**DOI:** 10.1002/advs.202517092

**Published:** 2026-01-18

**Authors:** Aradhana Dwivedi, Jan Dellith, Anna Makarova, Samir F. El‐Mashtoly, Juergen Popp, Vladimir Sivakov, Dana Cialla‐May

**Affiliations:** ^1^ Leibniz Institute of Photonic Technology Member of Leibniz Health Technologies Member of the Leibniz Centre For Photonics in Infection Research (LPI) Jena Germany; ^2^ Helmholtz‐Zentrum Berlin für Materialien Und Energie Berlin Germany; ^3^ Institute of Physical Chemistry (IPC) and Abbe Center of Photonics (ACP) Member of the Leibniz Centre For Photonics in Infection Research (LPI) Friedrich Schiller University Jena Jena Germany

**Keywords:** point‐of‐care platform, sensitivity, Silver Dendrites (AgDs), silver sulfate, stability, Surface‐Enhanced Raman Spectroscopy (SERS), therapeutic drug monitoring

## Abstract

Silver‐based surface‐enhanced Raman spectroscopy (SERS) substrates offer exceptional signal enhancement but suffer from poor long‐term stability due to oxidative degradation, limiting their utility for real‐world applications. Here, we introduce sulfate‐directed silver dendrites (s‐AgDs) as oxidation‐resistant SERS substrates with extended shelf life, reproducible signal performance, and sub‐picomolar sensitivity. Sulfate ions promote anisotropic growth and form a surface‐associated sulfate layer, resulting in dendritic morphologies with high hot‐spot density and suppressed surface oxidation. The AgDs demonstrate robust SERS performance over seven months under ambient conditions. We demonstrate translational utility by detecting five clinically relevant drugs in aqueous and blood plasma matrices with minimal pretreatment and excellent reproducibility. These results establish s‐AgDs as a stable, reproducible, and application‐ready SERS platform for point‐of‐care therapeutic drug monitoring.

## Graphical abstract

This study presents sulfate‐directed silver dendrites (s‐AgDs) as oxidation‐resistant SERS substrates with long‐term stability, reproducibility, and sub‐picomolar sensitivity. Sulfate ions guide anisotropic growth and protect the surface, enabling improved performance over 7 months under ambient conditions. The s‐AgDs detect multiple therapeutic drugs in blood plasma, enabling them as a robust platform for point‐of‐care drug monitoring.

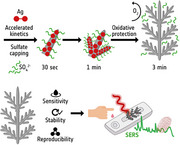

## 1 Introduction

Monitoring drug concentrations in a narrow therapeutic window is crucial for critically ill patients as they experience highly variable pharmacokinetics and reduced response to standard treatments [1, 2]. This prompts clinicians to use personalised therapeutic drug monitoring (TDM) to optimise dosing [3, 4]. Current state‐of‐the‐art TDM techniques include high‐performance liquid chromatography (HPLC) [5], liquid chromatography‐mass spectrometry (LC‐MS) [6], and immunoassays [7]. Although these methods are analytically rigorous, they require extensive sample preparation, trained personnel, and complex instrumentation; immunoassays additionally risk cross‐reactivity with drug metabolites. Consequently, there is an obvious demand for faster, easier, and real‐time TDM sensor platforms that can be used at the bedside.

Surface‐enhanced Raman spectroscopy (SERS) can amplify weak Raman signals by orders of magnitude through the plasmonic excitation of metallic nanostructures [8, 9, 10]. A compact Raman system combined with optimised sample preparation makes SERS highly attractive for POC testing [11, 12, 13]. SERS is widely utilised for the detection of clinically relevant biomolecules and biomarkers at trace amounts [14, 15]. It is a promising tool in TDM for many drugs, due to the presence of aromatic chemical structure, high polarizability, and strong metal‐molecule interaction [16, 17, 18]. Numerous studies have successfully monitored antibiotics [19], chemotherapeutic drugs [20], and metabolites [21, 22] with clinical relevance. However, to bring SERS into point‐of‐care use, it is necessary to overcome the challenges related to reliability, reproducibility, and long‐term stability of the SERS substrates.

Silver typically provides stronger SERS enhancement than gold, but its highly oxidative nature leads to degradation of performance over time [23]. This limitation often makes gold more preferred for biomedical applications. However, research continues to drive into methods of stabilizing silver substrates [24]. Multiple strategies are designed to protect silver from oxidation, including capping agents, deposition of thin oxide layers (e.g., SiO_2_, Al_2_O_3_) [25, 26], or protecting with biomolecules such as BSA [23]. These methods tend to reduce SERS efficiency by minimising metal‐molecule interactions. The shape and morphology of nanostructures critically influence the SERS efficiency [14]. Dendritic nanostructures offer high SERS enhancement due to their multibranched fractal‐like geometry, with numerous hot spots [26]. Silver dendrites (AgDs) are successfully used for SERS applications in the detection of pesticides [27], pollutants [28], organic molecules [29], and illicit drugs [30]. Very recently, it was introduced into wearable SERS sensors for analysing biomarkers in sweat, showing a great potential for non‐invasive monitoring of diabetes and lung cancer [31]. Nevertheless, the primary obstacle to commercial deployment remains the loss of performance within days. Clearly, there is a need for oxidation‐resistant designs that can protect the surface while maintaining plasmonic behaviour.

Here, we address this drawback by switching the precursor from AgNO_3_ to Ag_2_SO_4_ to produce sulfate‐stabilised silver dendrites (s‐AgDs) in a single growth step under 3 min. To our knowledge, only two papers have been published on AgD growth in the presence of SO_4_^2−^ ions. However, surface stoichiometries and robust SERS performance have not yet been investigated [32, 33]. A summary of the recently reported AgD substrates, with sensitivity and applicability, is provided in Table S1 for comparison with the present work. Sulfate ions passivate the surface, preserve SERS activity, and provide longer shelf life, as illustrated in Schematic 1. The SO_4_^2−^ passivation layer was confirmed by high‐resolution synchrotron radiation XPS and Raman spectroscopy. Stability tests revealed robust SERS performance for up to seven months. Signal variation remained below ±8% for spot‐to‐spot, inter‐batch, and batch‐to‐batch comparisons, and the detection limit was in the sub‐picomolar range. Finally, we tested five structurally unique drugs in both aqueous and blood plasma matrices. Even in the complex plasma matrix, clinically relevant low detection limits were reached, highlighting their translational potential. Overall, we developed a robust, oxidation‐resistant SERS platform that delivers reliable analytical performance for several months. We envisage that our study can open the way to next‐generation, POC platforms for reliable biomolecular detection.

## 2 Results and Discussion

### 2.1 Morphology and Growth Mechanism of AgDs

**SCHEME 1:**
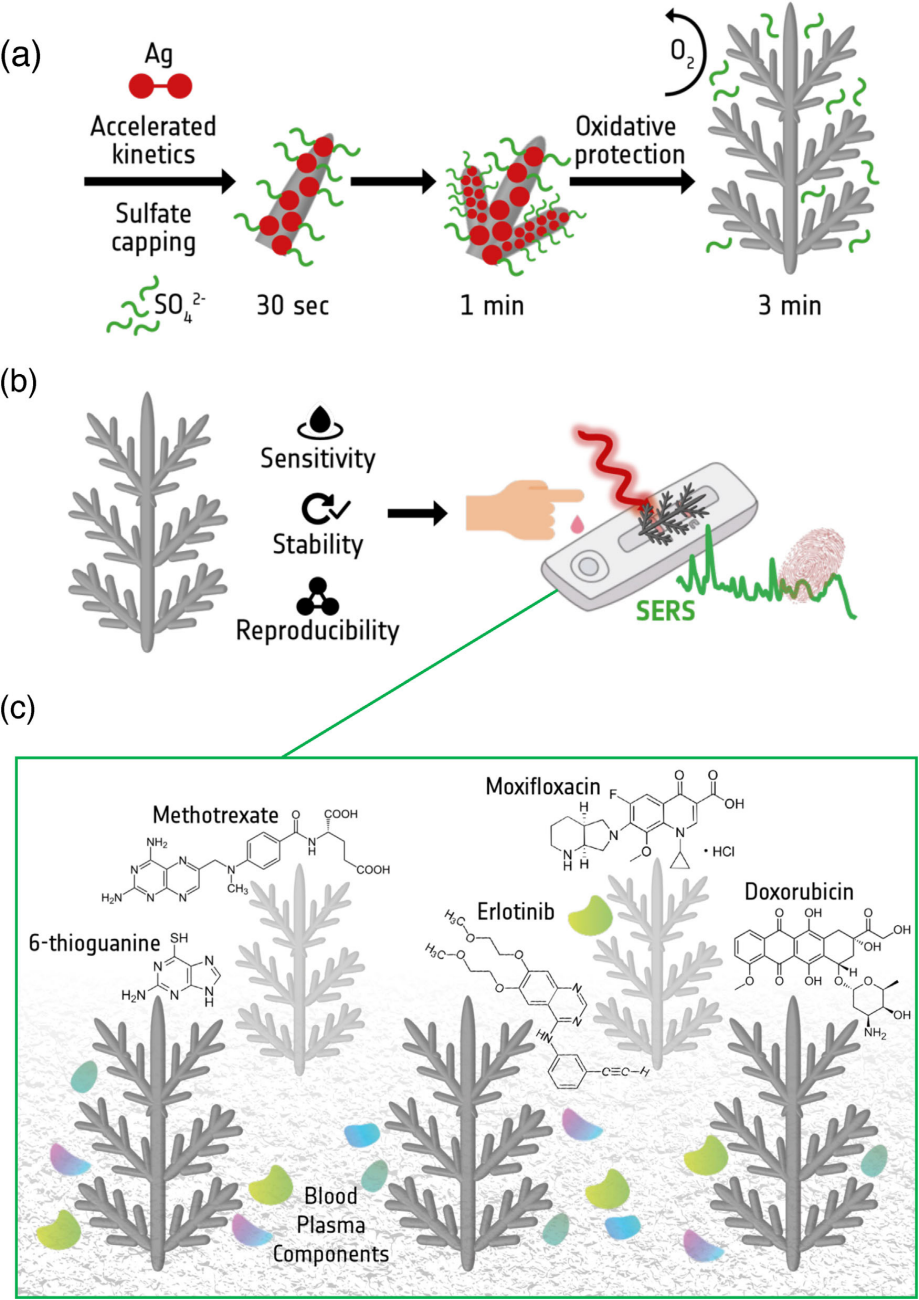
(a) Schematic illustration of rapid AgD growth via silver sulfate, (b) Conceptual scheme of SERS‐based POCT drug monitoring, (c) The five target drugs detected in the blood plasma showcasing the potential of AgDs.

We synthesized AgDs via a galvanic replacement between silicon (Si) and silver sulfate (Ag_2_SO_4_), replacing the conventional silver nitrate (AgNO_3_) precursor (Figure S1). This reaction occurs in an HF environment that promotes multibranched structure formation within ∼2.5 min (Figure S2) [34, 35, 36, 37]. Under HF conditions, Si oxidizes, releasing 4e^−^ to reduce Ag^+^ into metallic silver. Ag_2_SO_4_ provides a higher Ag^+^ flux and a sulfate environment that favors anisotropic dendrite growth (Scheme 1a), whereas AgNO_3_ yields slower growth and compact aggregates due to nitrate‐associated side reactions. To further confirm that dendrite formation is specific to Ag_2_SO_4_ and not a generic outcome of silver salt reduction, we compared multiple precursors under identical conditions (Figure S3); only Ag_2_SO_4_ yielded multibranched dendrites, while other anions produced compact aggregates.

**FIGURE 1:**
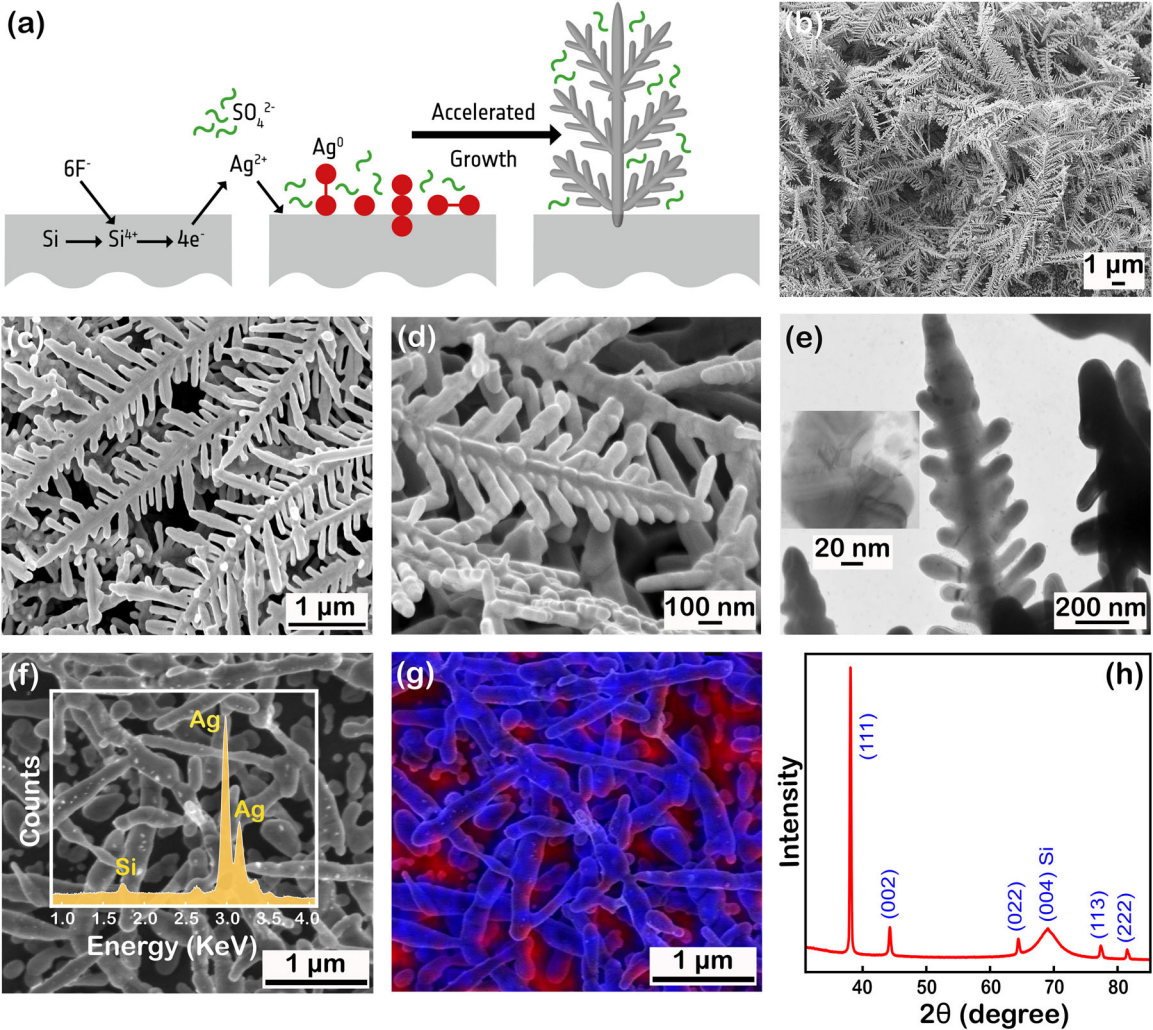
(a) Schematic of the galvanic growth mechanism for AgDs. (b–d) SEM images at increasing magnification. (e) TEM image of a dendritic branch and an inset showing some grain boundaries. (f) SEM‐EDX spectrum. (g) EDX elemental mapping showing Ag (blue) and the Si (red) distribution. (h) XRD pattern of AgDs compared with the references (98‐006‐1079; Silver; Aq1; Cubic, 98‐002‐2988; Silicon; Si1: Cubic).

Scanning electron microscopy (SEM) images (Figure 1b–d) revealed a well‐defined multibranched dendritic morphology with extensive surface roughness conducive to enhancing local electromagnetic fields for SERS activity. From transmission electron microscopy (TEM) (Figure 1e), the structure appears homogeneous with only a few grain boundaries (inset) in the dendrite branches. Elemental analysis via energy‐dispersive X‐ray spectroscopy (EDX) (Figure 1f,g) further revealed the high silver content across the dendrite structures.

X‐ray diffraction analysis (XRD) pattern (Figure 1h) confirmed a face‐centered cubic (FCC) crystal structure with dominant (111) reflection, consistent with primary branch growth. The rounded tips and branched morphologies observed in the SEM and TEM images (Figure 1d,e) are characteristic of kinetically favoured growth. Under such conditions, rapid formation of high‐energy surfaces promotes rounded tip elongation, while limited time for atomic rearrangement surpasses the formation of equilibrium favoured shapes such as polyhedral nanocrystals [38]. In contrast, longer reaction times lead to the formation of faceted geometries at the tip end associated with the thermodynamically stabilized morphologies. The dendritic structures observed here are consistent with rapid growth under kinetic control, as evident from the short synthesis duration during our experiments. The full dendrite formation is achieved within ∼2.5 min, as illustrated in Figure S2.

The evolution process of dendrites is governed by the interplay between thermodynamic and kinetic factors [35]. During early‐stage nucleation under high supersaturation, growth proceeds under non‐equilibrium conditions, leading to the diffusion‐limited aggregation (DLA) and tip‐focused deposition [35]. The newly generated atoms preferentially accumulate at the high‐energy sites, initiating growth in the (111) direction to form primary branches, followed by secondary and tertiary branches. Over time as the local atomic concentration declines, growth may gradually transition toward thermodynamic regimes, favouring faceted nanostructures [39]. However, when metal atoms are generated at a high rate, the resulting structures are compelled to adapt to the kinetically dominated shape, in this case, dendrites. Due to higher silver ion availability from Ag_2_SO_4_ compared to AgNO_3_, owing to its two to one stoichiometry, it sustains a steep concentration gradient that reinforces kinetically driven dendrite growth as illustrated schematically in Figure S4.

As is well established in the literature, electroless galvanic displacement on silicon in HF yields AgDs [36, 37]. In our approach, we replace the conventional AgNO_3_ with Ag_2_SO_4_, which enables better growth control and rapid AgDs formation without the need for elevated temperatures or high precursor concentrations. The reaction is localised and self‐limiting, forming seed sites that extend further through the DLA process. The interplay between the primary seeds, the rate of availability of Ag atoms, and the DLA dynamics governs the final length, complexity, and branching order of the dendrites [40]. This one‐pot, room‐temperature process is completed under 3 min and is compatible with wafer‐scale fabrication and facile dicing into individual SERS chips, offering a straightforward industrial scale‐up possible.

### 2.2 Sulfate‐Associated Surface Chemistry

As illustrated in the previous sub‐chapter, replacing AgNO_3_ with Ag_2_SO_4_ accelerates silver growth many‐fold, and here we will discuss in detail the role of SO_4_^2−^ ions. Apart from silver ion concentration and reaction time, the type of co‐existing anion plays a critical role in growth behaviour and final morphology. Anions such as F^−^, SO_4_^2−^, and Cl^−^ are known to influence the crystal growth without becoming part of the crystal lattice [32]. These ions, whether present in the precursor or added as foreign ions, can either promote or inhibit specific crystal facets or modify redox kinetics, thereby accelerating or decelerating the synthesis process.

It was observed that silver growth was much slower with AgNO_3_, resulting in aggregates (AgA) with reduced 3D morphology. This is shown in Figure S5, which shows that AgNO_3_‐derived AgAs formed after 5 min, in contrast to the complete dendrite formation achieved using Ag_2_SO_4_ within just 2.5 min (Figure S2). Reduced structural complexity and density of plasmonic hotspots resulted in lower SERS sensitivity, with the lowest detected concentration being 10^−8^ m on AgAs. A comparison with 10^−13^ m on AgDs is demonstrated in Figure S9. These differences were confirmed by SEM, EDX, and SERS analyses (Figure S5). Besides differences in Ag^+^ stoichiometric factor in the chemical formulas, 1 (AgNO_3_) and 2 (Ag_2_SO_4_), the type of anion (NO_3_^−^ vs. SO_4_^2−^) is also influential. Nitrate can accept electrons under acidic conditions, readily participate in redox reactions, take electrons from Si to form the byproducts (NO, NO_2_), and SiO_2_ on the surface [41]. This additional oxide layer reduces electron transfer efficiency and lowers the overall reduction rate (Ag^+^ to Ag^0^). In contrast, SO_4_^2−^ ions are redox inactive at room temperature [42], allowing full electron flux to be utilized in Ag^+^ reduction, and increases growth rate. It has already been studied that the formation and density of dendrites increase at higher SO_4_^2−^ concentrations, whether introduced as a foreign substance or present in the precursor form [32, 33]. Sulfate ions also influence both the nucleation and growth stages by acting as ionic surfactants. They selectively adsorb onto high‐energy crystal facets, modulating surface energies and promoting anisotropic growth [32, 33]. It is hypothesised that SO_4_^2−^ co‐adsorbs during the early nucleation stage and remains associated with the growing surface, directing the deposition process via DLA [32].

**FIGURE 2:**
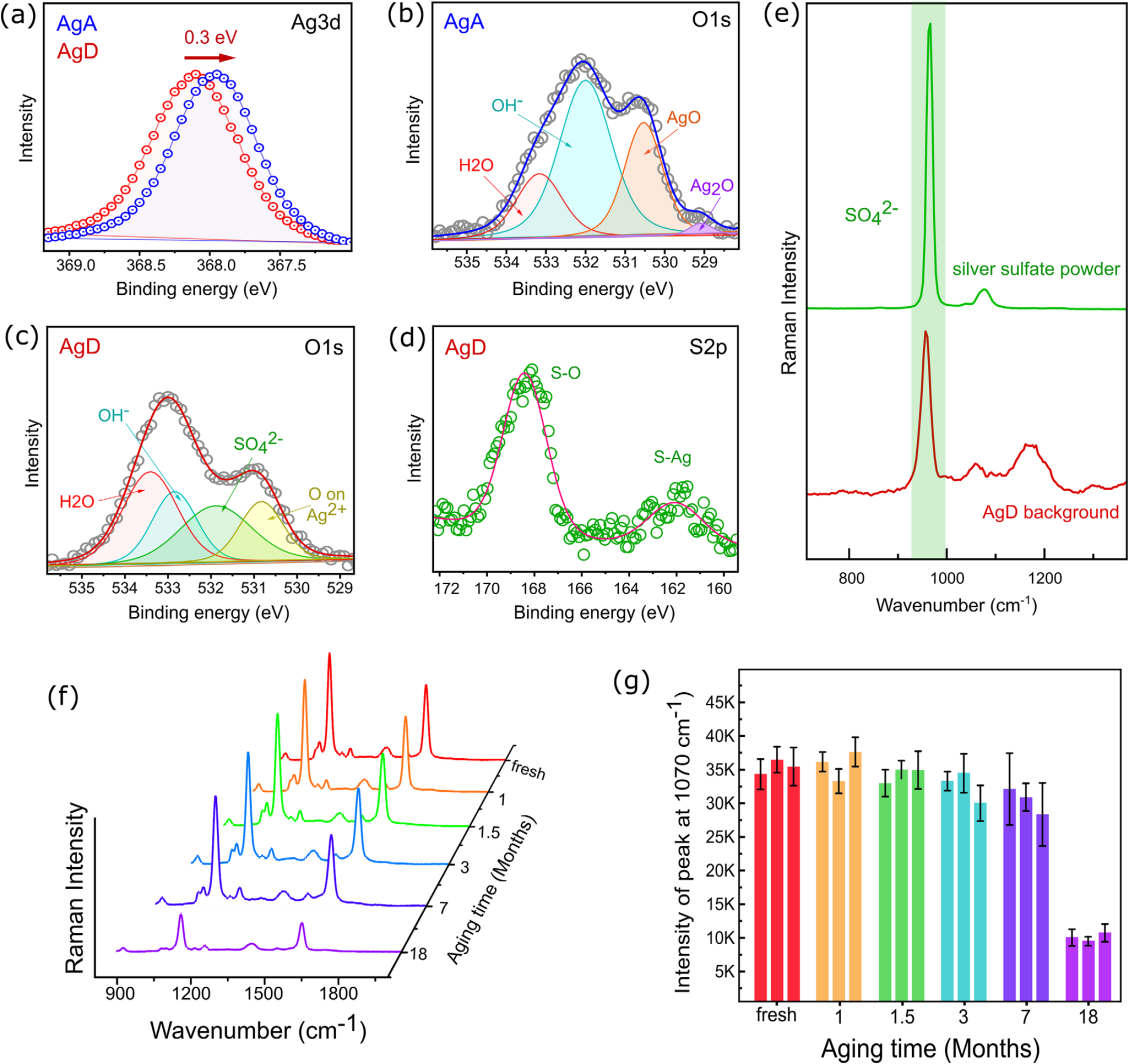
XPS spectra of (a) Ag 3d. (b) O1s for n‐AgAs. (c) O1s for s‐AgDs. (d) S2p for s‐AgDs (e) Raman spectra comparing Ag_2_SO_4_ powder and s‐AgDs. (f) Mean SERS spectra of s‐AgDs s at different aging times with 4‐MBA 10^−5^ m; for each aging time, n = 3 substrate replicas and 50 spectra per substrate. (g) Average intensities at 1070 cm^−1^ with standard deviations for each aging time, measured across n = 3 substrate replicas and 50 spectra per substrate.

X‐ray photoelectron spectroscopy (XPS) further validated it in the surface stoichiometry. In s‐AgDs, the Ag 3d5/2 peak appeared at 368.1 eV, showing a slight (0.1 eV) shift from the metallic Ag reference (368.2 eV), indicates predominantly Ag° character (Figure 2a). Conversely, nitrate‐derived aggregates (n‐AgAs) showed an evident 0.3 eV shift (to 367.9 eV), suggesting surface oxidation and formation of species such as AgO/Ag_2_O. The O1s spectrum of n‐AgAs (Figure 2b) revealed dominant peaks for Ag_2_O (529 eV), AgO (530.5 eV), surface OH groups (532 eV), and adsorbed H_2_O (533.2 eV), consistent with the increased surface oxidation [43, 44, 45]. In s‐AgDs, the O1s spectrum (Figure 2c) showed peaks at 530.6 eV corresponding to oxygen on Ag^2+^, oxygen from SO_4_^2−^ (531.6 eV), O on OH^−^ (532.6 eV), and adsorbed H_2_O (533.2 eV) [44]. The S2p spectrum of s‐AgDs (Figure 2d) resolves two doublets: The low binding energy component is related to the sulfur atoms bonded to silver (S‐Ag:162 eV), while the high binding energy one refers to oxides (S─O:168.4 eV) [46], thus providing further validation of the chemisorption of SO_4_^2−^ as a passivating layer. Raman spectra also confirmed the presence of SO_4_^2−^ at the s‐AgDs surface. It is important to note that all XPS spectra in Figure 2a–d were acquired from one‐month‐old substrates, demonstrating that sulfate species remain stably associated with the surface beyond initial synthesis. A distinct vibrational mode at ∼970 cm^−1^ (Figure 2e) is attributed to SO_4_^2−^ Raman mode [47, 48], which is also clearly present on the AgD surface and is consistent with the XPS results confirming the presence of sulfate species. A slight red shift of this mode in s‐AgDS compared to powder Raman spectra of Ag_2_SO_4_ might be due to a bonding change between Ag and SO_4_^2−^. The results show that SO_4_^2−^ has two functions: it accelerates the AgD growth by increasing the rate of electron transfer, and further, passivates the surface. This combined effect improves the structural definition and environmental resilience of s‐AgDs.

From the perspective of the SERS enhancement mechanism, the sulfate species detected by XPS indicates a sulfate terminated surface, not a thick sulfate layer. The SO_4_^2−^ are loosely bound to Ag, functioning as a dynamic ionic surfactant that passivates the surface against oxidation but are readily displaced when analyte molecules enter the hotspots. Because the LSPR of silver dendrites is intrinsically broad and morphology‐dominated, such a thin, discontinuous termination is not expected to cause a resolvable resonance shift. Conversely, thicker, continuous oxide or polymer coatings (or strong organic capping) are known to cause clear LSPR shifts and influence charge transfer directly [49]. More broadly, our s‐AgDs demonstrate LSPR‐dominated enhancement alongside interfacial durability and reproducibility. This is in line with advances in stable optical sensing platforms, where geometric and surface control ensure long‐term sensitivity and enhance Raman and related optical signals [50, 51].

## 3 Analytical Performance of s‐AgDs

### 3.1 Stability

To translate the surface passivation by SO_4_^2−^ into real‐world performance, we monitored the SERS activity of s‐AgDs for up to 18 months. Substrates were synthesized according to the standard protocol and stored at ambient conditions. The SERS performance was evaluated at six‐time intervals: freshly prepared, and aged for 1, 1.5, 3, 7, and 18 months (Figure 2f,g). The aged substrates were incubated in 4‐MBA solution (10^−5^ m) for 10 min, prior to the SERS measurement. For each condition, three replicate substrates were analysed, and 50 spectra were collected per substrate to ensure statistical significance. The average SERS spectra shown in Figure 2f reveal the excellent signal retention between freshly prepared, 1‐month old, and 1.5‐months old samples, with a marginal decline after 3 months. By seven months, a moderate reduction in the SERS intensity and an increase in the variability were observed, which may be due to the partial degradation of the surface. After 18 months, the SERS signal was significantly reduced, likely due to environmental contamination and degradation of the surface over time. The stable signal retention of s‐AgDs for up to seven months of shelf storage confirms that SO_4_^2−^ passivation improves the durability of silver. To complement the functional SERS stability, we compared S 2p XPS spectra of freshly prepared and one‐month‐old AgDs, which confirm that surface‐bound sulfate species persist over time (Figure S10). This extended stability highlights their potential for real‐world applications where substrate longevity and minimal user maintenance are critical.

### 3.2 Sensitivity

**FIGURE 3:**
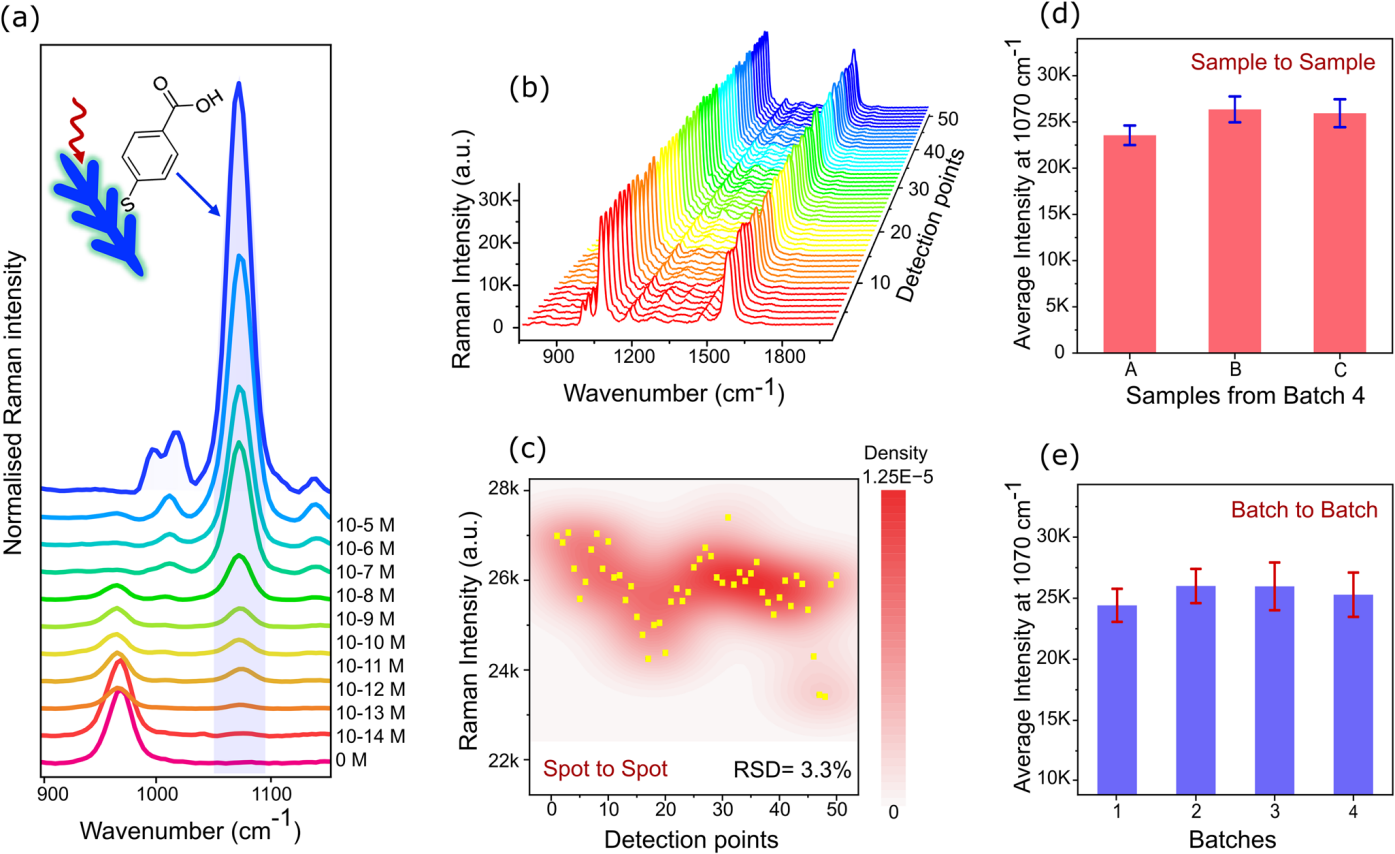
(a) Average SERS spectra of 4MBA (10^−5^ to 10^−14^ m concentration (n = 3 substrate replicas per concentration, 5 spectra per substrate; 15 spectra averaged per concentration); inset: schematic of 4‐MBA attached to the AgD surface. (b) SERS spectra from 50 different spots on a single s‐AgD substrate. (c) Corresponding intensity distribution of 1070 cm^−1^ mode for the 50 spots shown in (b). (d) Sample‐to‐sample reproducibility within one batch (batch 4) for three substrate replicas (A–C; n = 50 spectra per substrate), shown as mean intensities at 1070 cm^−1^ ± SD. (e) Batch‐to‐batch reproducibility for four independent batches (n = 3 substrates per batch, 50 spectra per substrate), shown as mean intensities at 1070 cm^−1^ ± SD.

To measure the sensitivity of s‐AgDs, 4‐MBA was used as the probe molecule. All s‐AgDs were incubated for 10 min in aqueous 4‐MBA solutions prepared at concentrations ranging from 10^−5^ to 10^−14^ m. For each individual concentration, three replicates were used with 5 spectra per replicate. The final spectra presented in Figure 3a show the averaged signal from 15 spectra per concentration, providing statistical reliability. Individual mean spectra for 3 batches are provided in Figure S6. The results demonstrate a clear and consistent detection of characteristic 4‐MBA peaks at 1070 and 1590 cm^−1^, extending down to 10^−13^ m. This establishes the LOD as 0.49 pM and the LOQ as 0.92 pM, calculated from the log‐log calibration curve (Figure S7). At ultra‐low concentrations (∼10^−11^–10^−13^ m), the SERS intensity decreases, and minor peak shifts/line‐shape changes are observed, which we attribute to monolayer or sub‐monolayer adsorption regimes and orientation effects at low surface coverage. Below this level, the signal reproducibility was compromised owing to the stochastic nature of analyte distribution. At such low concentration levels, uniform molecular distribution across the surface is highly unlikely, and signal averaging across the surface may underestimate actual detection capability due to contributions from analyte‐free regions. The inherent background signal of the substrate at 970 cm^−1^ originates from the surface‐bound SO_4_^2–^ ions. This peak is prominently observed at low analyte concentrations due to the limited surface coverage of 4‐MBA. With increasing concentrations, the SO_4_^2–^ signal gradually decreases and eventually disappears, owing to competitive adsorption, as 4‐MBA molecules increasingly occupy the SERS‐active sites. This displacement highlights the dynamic surface interaction on the AgDs surface and validates the gradual enrichment of the analyte signal. Reliable sub‐picomolar SERS sensitivity of s‐AgDs makes them attractive for low‐ abundance analyte quantification in biomedical assays.

To quantitatively benchmark substrate performance, we calculated the analytical enhancement factor (AEF) for 4‐MBA following established procedures [52, 53] by comparing the SERS intensity on Ag dendrites with the Raman intensity measured on bare Si under identical measurement conditions. Using the 4‐MBA band at 1070 cm^−1^, the AEF values were evaluated at the four lowest detectable concentrations (10^−10^–10^−13^ m) to provide a robust and internally consistent benchmark of enhancement in the ultrasensitive regime and to mitigate potential overestimation associated with single‐concentration reporting. The AEF values, ranging from 2.2 × 10^8^ to 4.5 × 10^10^, provide a quantitative benchmark for the AgDs substrate. The reference spectrum, calculation details, and the AEF summary table are provided in Figure S8, Table S2.

### 3.3 Reproducibility

To quantify both intra‐batch (point‐to‐point and sample‐to‐sample) and inter‐batch variability, a total of 600 spectra were collected over 50 spots, with 3 replicates and 4 independent batches. Single‐point measurements were conducted using a 10^−5^ m 4‐MBA as a probe. Spectra were collected from 50 distinct locations across each substrate. To strengthen the statistical reliability of the analysis, four independent batches were prepared, each comprising three substrate replicates (A, B, and C). Altogether, 50 spectra from each replicate, making a total of 150 spectra per batch, were acquired from unique positions across the substrates. Reproducibility was assessed across three levels: point‐to‐point, sample‐to‐sample, and batch‐to‐batch.

Point‐to‐point reproducibility is presented in Figure 3b,c for an individual substrate (replicate B from batch 2), showcasing the SERS spectra and the corresponding intensity distribution of the 1070 cm^−1^ peak. An RSD of 3.3% was observed, indicating excellent local uniformity. Sample‐to‐sample reproducibility was analyzed by comparing 3 replicates from batch 4 (Figure 3d), which showed RSD values of 4.5%, 5.3%, and 5.9%, respectively, illustrating the consistent performance of substrates. Detailed reproducibility data for all 12 replicas across the four batches are provided in Figure S11. Finally, batch‐to‐batch reproducibility was assessed by comparing the average SERS intensities from all four batches (Figure 3e), yielding RSD values of 5.5%, 5.4%, 7.5%, 7.2%, respectively. In all cases, the RSD values remained below 10%, demonstrating good consistency among various synthesis batches. The RSDs of 3%–6% within batches and ≤ 8% between batches show that s‐AgDs provide a reliable SERS signal, which is necessary for quantitative bioanalytical workflows in regulated clinical settings. To visualize spatial signal heterogeneity, representative SERS areamaps were also tested across different substrate regions (Figure S12). Specifically, three Raman maps were acquired (Figure S12): 10 × 10 µm^2^ on one substrate, and 10 × 10 µm^2^ plus 20 × 20 µm^2^ on a second substrate, using the 4‐MBA band at 1070 cm^−1^, yielding RSD = 14.46 ‐15.91% across all pixels. These maps and the corresponding spectral stacks confirm a spatially consistent microscale SERS response, while the remaining local intensity fluctuations are consistent with expected variations on 3D dendritic architectures (e.g., local hotspot density, laser focus variations on uneven topography, and molecular distribution).

## 4 Application to TDM in Human Plasma

To test the clinical relevance of the s‐AgDs for TDM, we evaluated a diverse panel of clinically relevant drugs in both aqueous and human plasma matrices. Selected drugs, 6‐thioguanine, erlotinib, methotrexate, doxorubicin, and moxifloxacin, represent distinct chemical structures, varied surface interaction characteristics, and spectral fingerprints. Each selected drug carries unique significance. For instance, 6‐Thioguanine is an anticancer drug used in the treatment of breast cancer and leukemia, but its excessive use can lead to severe side effects [54]. Erlotinib is a tyrosine kinase inhibitor that is used in the treatment of lung cancer. It has been reported to have side effects that include skin rash and diarrhea in patients [55]. Methotrexate is one of the most common chemotherapeutic drugs administered for acute lymphoblastic leukemia (ALL), resulting in toxic effects from elevated blood levels [56]. Doxorubicin is widely used in the treatment of various human malignancies, is highly cardiotoxic, and demands strict dosage control [57]. Finally, moxifloxacin is an antibiotic prescribed for respiratory tract infections; achieves rapid systemic absorption, needing reliable monitoring shortly after oral administration [58]. Although TDM requires clinically guided dose adjustments based on pharmacokinetic profiling and target plasma levels, the focus of this study is to demonstrate the analytical suitability of our substrates for such applications, rather than conducting clinical TDM itself.

**FIGURE 4:**
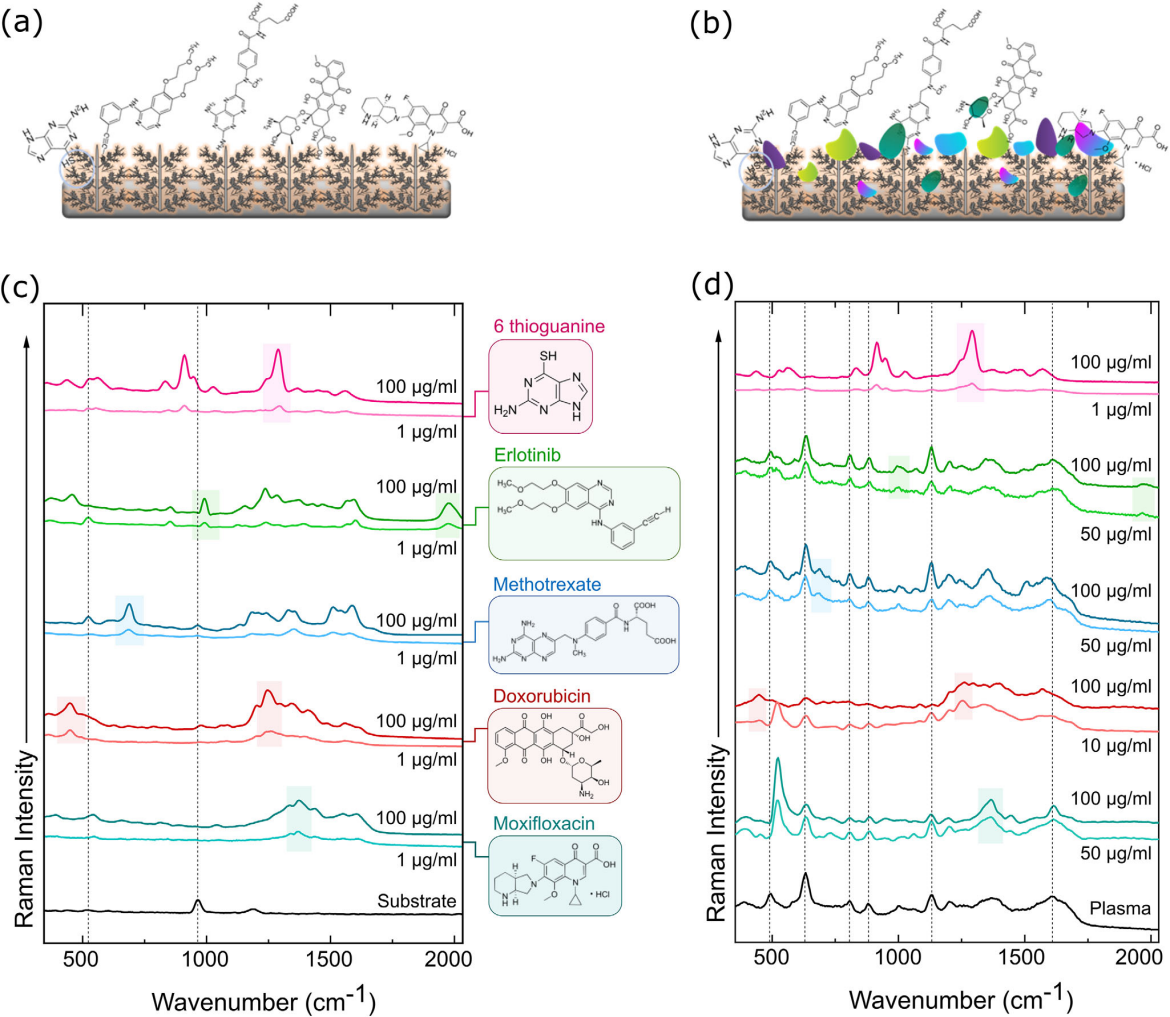
Schematic representation of drug interactions in (a) an aqueous environment and (b) a complex blood plasma matrix. (c) SERS spectra of five drug molecules at 1 and 100 µg/mL, along with the background spectrum from the substrate in an aqueous environment. (d) SERS spectra of the respective drugs spiked into a blood plasma matrix at 100 µg/mL, along with the lowest detectable concentration.

An initial SERS test was conducted in aqueous solution (Figure 4a), at 1 and 100 µg/mL concentrations, as the therapeutic window for most of the drugs falls between these concentrations (Figure 4c). Despite the presence of SO_4_^2−^ on the surface, no significant signal interference was observed. Each drug exhibited their unique and clearly identifiable spectral fingerprints at both tested concentrations, confirming the suitability of the s‐AgDs for trace‐level SERS‐based drug detection. To simulate realistic clinical scenarios, the same drugs were spiked at identical concentrations into untreated, undiluted human blood plasma (Figure 4d), significantly increasing the complexity of the analytical matrix (Figure 4b). Although the SO_4_^2−^ background had minimal to no interference with the drug spectra, a strong plasma background was apparent at the lower tested drug concentration (1 µg/mL). Only 6‐thioguanine retained clearly identifiable spectral features down to 1 µg/mL, likely due to its favorable interaction with the s‐AgDs. In contrast, doxorubicin was reliably detected down to 10 µg/mL, whereas erlotinib, methotrexate, and moxifloxacin exhibited higher detection thresholds of 50 µg/mL, likely due to weaker interactions or stronger spectral interference from background plasma matrix. This matrix interference underlines the necessity of a plasma sample pretreatment step to maximize detection sensitivity at low concentration clinical detection. However, these data also indicate that when a drug possesses a favorable interaction, our s‐AgDs can be effectively applied directly in untreated plasma samples, offering an immediate and simplified approach for TDM in complex biological matrices.

To enable improved detection of the target drugs at lower concentrations, a blood plasma sample preparation method was applied, as previously reported by our group [59, 60], which uses acetonitrile‐based protein precipitation from plasma to effectively reduce background interference. This step underlines the importance of adequate sample processing in complex biological matrices for achieving high sensitivity and specificity during SERS‐based TDM. To comprehensively analyze the sensitivity and quantitative performance of s‐AgDs, the detection limit was evaluated in both aqueous solution and blood plasma. For each selected drug, a broad concentration series was prepared starting at 100 µg/mL down to the lowest reliably detectable concentration in both the matrices (Figure S13). The full spectral dataset, including aqueous and plasma matrices and their respective calibration curves are systematically presented in Figure S13 (a_1_‐a_5_ for aqueous, c_1_‐c_5_ for plasma, and b_1_‐b_5_ for the respective calibration curves). The LOD and LOQ values calculated from these calibration curves are summarized comprehensively in Table S3.

**FIGURE 5:**
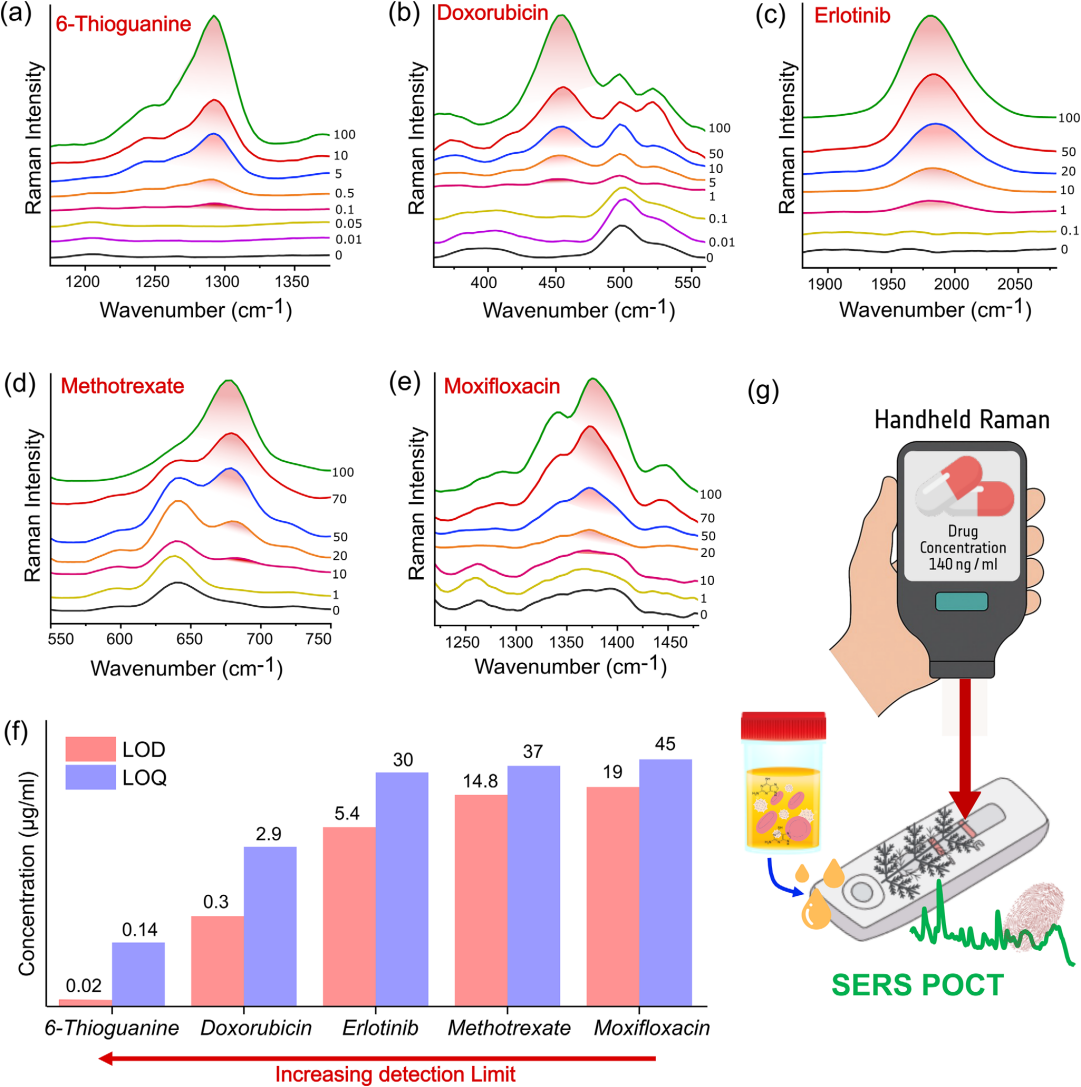
Average SERS spectra of the drugs spiked into and pretreated in a blood plasma matrix obtained from three independent sample batches (n = 3) for each analyte: (a) 6‐thioguanine, (b) doxorubicin, (c) erlotinib, (d) methotrexate, (e) moxifloxacin. (f) Respective limit of detection and quantification (LOD/LOQ) for each drug in the pretreated plasma. (g) Schematic representation of the potential integration of AgDs to develop an effective POC device.

Figure 5a–e shows only the distinct, interference‐free characteristic peaks for each drug in the treated plasma matrix (red shaded peak areas). This highlights the spectral markers used for quantitative analysis, which are critical for practical applications, ensuring clarity and immediacy for the reader. These marker modes were selected for quantitative analysis due to their optimal signal‐to‐noise ratios, minimal spectral overlap with plasma background signals, and strong reproducibility across three replicate batches in both aqueous and plasma matrices. Figure 5f further summarizes these data by presenting the LOD and LOQ values in a concise bar plot that clearly illustrates the sensitivity trends among the five tested drugs. This visual summary allows for instant comparison of the detection capabilities of s‐AgDs in actual clinical settings. It also highlights the wide range of possibilities for drug‐specific detection on the dendrite surface.

From Figure 5f, the lowest detection limit was observed for 6‐thioguanine (0.02 µg/mL), benefiting from a favorable thiol‐metal interaction. Doxorubicin (0.3 µg/mL) ranked second likely due to its high intrinsic Raman cross‐section, closely followed by erlotinib (5.4 µg/mL), possibly due to its distinct alkyne mode in the Raman silent region that minimizes spectral overlap with plasma components. Methotrexate (14.8 µg/mL) displayed a higher detection limit, possibly influenced by its pH‐dependent absorption characteristics, which was avoided in this study to maintain methodological consistency. Moxifloxacin (19 µg/mL) showed the highest detection limit, likely due to its structural complexity and limited interaction with the metallic surface. These findings show that leveraging drug‐specific chemical and structural properties, combined with a simple protein removal step, can significantly improve the practical applicability of the s‐AgDs for a robust and sensitive POCT platform for real‐time TDM in complex biological matrices.

Lastly, Figure 5g conceptually illustrates the potential application of the developed s‐AgDs within a POC workflow. Integrating disposable s‐AgDs within a simple POC cassette accompanied by a handheld Raman spectrometer offers a robust platform for bedside drug detection and identification. This integration leverages the analytical robustness, stability, and reproducibility of s‐AgDs to establish a foundation for next‐generation POC systems.

## 5 Conclusion

In this study, we present a rapid and robust approach to fabricate sulfate‐mediated silver dendrite (s‐AgD) substrates by a simple precursor switch from AgNO_3_ to Ag_2_SO_4_. Sulfate ions play a dual role at the nanoscale, directing the formation of multibranched dendritic structures while simultaneously passivating the surface to prevent oxidation. This significantly extends stability and shelf life. Consequently, these s‐AgDs exhibit outstanding plasmonic stability, as demonstrated by their sustained SERS performance for at least seven months under ambient storage conditions. Furthermore, these s‐AgDs demonstrated strong analytical performance with sub‐picomolar sensitivity and inter‐ and intra‐batch reproducibility consistently below 8%. To demonstrate the practical applicability of these s‐AgDs in bioanalytical applications, we successfully showed that they can be used for sensitive TDM of five unique drugs in aqueous and blood plasma matrices. Some drugs (6‐thioguanine and doxorubicin) were even detected at trace concentrations in undiluted plasma. A simple protein precipitation step reliably quantified all five chemically diverse drugs (6‐thioguanine, 0.02 µg/mL; doxorubicin, 0.3 µg/mL; erlotinib, 5.4 µg/mL; methotrexate, 14.8 µg/mL; and moxifloxacin, 19 µg/mL) in human blood plasma. This high analytical performance, even in the presence of a complex plasma background, highlights the strong potential of s‐AgDs for use in real clinical scenarios. Together, these results position s‐AgDs as a stable, reproducible SERS platform suitable for translational therapeutic drug monitoring.

Looking forward, the reported sulfate‐assisted stabilization strategy could potentially be extended to other oxidation‐prone plasmonic metals (e.g., Cu, Al) for controlled growth and longer shelf life. Coupled with handheld Raman instrumentation and machine‐learning‐assisted spectral interpretation, s‐AgDs could underpin next‐generation, portable POC diagnostics. More broadly, this work illustrates how nanoscale growth control and surface chemistry can address long‐standing stability barriers in SERS, advancing reliable, clinically relevant Raman analytics.

## 6 Methods

### 6.1 Materials

Acetonitrile (CAS Nr. 75‐05‐8, ≥99,9 %), acetone (CAS Nr. 67‐64‐1, ≥99,8 %), and isopropanol (CAS Nr. 67‐63‐0, ≥99,8 %) were purchased from Carl Roth GmbH + Co. KG (Germany). Silicon wafers (p‐type boron‐doped) with a thickness of 525±25 µm and a diameter of 100 mm with a resistivity of <0.005 Ωcm were purchased from Si‐Mat—Silicon Materials e.K. (Germany). Silver sulfate (CAS Nr. 10294‐26‐5, ≥99.99%), silver nitrate (CAS Nr. 7761‐88‐8, ≥99.0%) 4‐mercaptobenzoic acid (CAS Nr. 1074‐36‐8, 99%), 6‐thioguanine (CAS Nr. 154‐42‐7, ≥98%), erlotinib hydrochloride (CAS Nr. 183319‐69‐9, ≥98%), methotrexate (CAS Nr. 59‐05‐2, ≥98%), doxorubicin (CAS Nr. 25316‐40‐9, 98.0‐102.0%), moxifloxacin (CAS Nr. 186826‐86‐8, ≥98%) and hydrofluoric acid (Cas Nr. 7664‐39‐3, 48%) were ordered from Sigma Aldrich (Germany). All reagent solutions were prepared with ultra‐high purity deionized water (resistance larger than 18 MΩ), unless otherwise stated.

Blood plasma obtained from a healthy volunteer was purchased from the Institut für Klinische Transfusionsmedizin Jena GmbH (Jena, Germany). The blood samples were purchased from the Institut für Klinische Transfusionsmedizin Jena GmbH (Jena, Germany). All methods were performed in accordance with the relevant guidelines and regulations of the clinics of Universitätsklinikum Jena; thus, the blood samples were exempt from the need for ethical approval [59]. The plasma samples were aliquoted into smaller volumes and stored at −18°C. Prior to each use, the aliquots were thawed and brought to room temperature.

Methotrexate stock solution (500 µg/mL) was dissolved into NaOH (0.1 m) + DI water, and erlotinib stock solution was prepared in ethanol (250 µg/mL) and diluted in DI water for lower concentrations. Stock solutions of 6‐thioguanine, doxorubicin, and moxifloxacin were prepared in DI water at a concentration of 1000 µg/mL and diluted further to achieve lower concentrations.

### 6.2 Methods

#### 6.2.1 SEM and EDX

The SEM measurements were performed with a field emission scanning electron microscope JSM‐6700F (JEOL, Tokyo, Japan). The electron energy was varied between 3 and 5 keV. To enhance the surface sensitivity and the topographical impression, some of the micrographs were taken at a stage tilt of 25 ‐ 45°. All Energy‐dispersive X‐ray spectroscopy (EDX) was done using a state of the art 100 mm^2^ silicon drift detector (SDD) by BRUKER (BRUKER Nano GmbH, Berlin, Germany) and the Esprit spectra evaluation software package. The specified energy resolution of the detector at 5.9 keV (Mn‐Kα) was 129 eV.

#### 6.2.2 TEM

TEM images were collected using a Hitachi HT7820 instrument (Hitachi High‐Tech Corporation, Tokyo, Japan) with an acceleration voltage of 100 kV and a 20 Megapixel bottom‐mount CMOS TEM camera Emsis Xarosa (Emsis GmbH, Münster, Germany). For this, AgDs were transferred on carbon coated Cu grids (Formvar filmed grids; Plano GmbH) followed by vacuum drying.

#### 6.2.3 XRD

The XRD analysis has been performed with an X′pert Pro MPD Instrument theta‐theta diffractometer (PANanalytical, Almelo, Netherlands) using Cu‐Kα_1,2_ radiation. For phase identification, the Malvern Panalytical HighScore Plus Software V.4.9 was used with databases ICDD, ICSD, and COD. The Scherrer equation was used for the determination of the crystallite sizes.

#### 6.2.4 XPS

High‐resolution XPS spectra were obtained at the electron storage ring BESSY II at Helmholtz Zentrum Berlin für Materialien und Energie (HZB) using the facilities of the GELEM‐PES dipole beamline [61, 62]. XPS spectra were acquired with a hemispherical Phoibos 150 electron energy analyzer (Specs GmbH) using a photon energy of 700 eV. For binding energy calibration, all spectra were aligned relative to the Au 4f_7/2_ core level peak of a reference gold single crystal, set to 84.0 eV binding energy. All measurements were carried out at room temperature. The base pressure was 1 × 10^−10^ mbar.

#### 6.2.5 Synthesis of Silver Dendrite Substrates

The silicon substrates were first cleaned in acetone and isopropanol, followed by DI water, to remove the impurities. After removing native oxide in 2% HF for 1 min, the substrates were transferred to the galvanic deposition bath. The bath was prepared by mixing equal volumes of 0.01 m silver sulfate (or silver nitrate) and 5 m HF. The deposition was allowed to proceed for 30 s to 5 min, respectively, after which the substrate was washed in DI water and dried under a gentle nitrogen stream. A schematic of the deposition procedure is shown in Figure S1.

#### 6.2.6 Blood Plasma and Pretreatment

The drugs were spiked into blood plasma by maintaining the ratio of 1:9 of drug solution: blood plasma. In this process, 20 µL of drug solution (for each tested concentration) was added to 180 µL of blood plasma to get the desired concentrations. The proteins were removed from the spiked plasma solution using acetonitrile‐based precipitation [59, 60]. Acetonitrile was added to the spiked plasma solution in the ratios of 1, 3, 5, 7, 9, and sonicated for 5 min. After that, it was kept for 10 more minutes and allowed for the proteins to be precipitated completely. This solution was centrifuged at 8000 rpm for 10 min, and the supernatant was used for SERS analysis.

#### 6.2.7 Raman and SERS Measurements

SERS measurements were carried out using a WITec alpha300R spectrometer (Ulm, Germany). The system was equipped with a 785 nm laser excitation line and an electron multiplying charge coupled device. A microscopic lens of 10x objective (Olympus) and 0.25 numerical aperture, and a grating of 300 grooves/mm was used. The incident laser power on the sample surface was 1 mW with an integration time of 1 s and 3 accumulations, unless stated otherwise. For each SERS‐based investigation, concentration‐dependent detection of 4‐MBA as well as of various drugs, three parallel batches, synthesized on three different days of the SERS substrates were used. And each batch consists of three substrate replicates, synthesized on the same day. For each substrate, 50 single spectra were randomly recorded, and the mean spectrum and the standard deviation were estimated accordingly. The silver dendritic substrates were incubated for 10 min in the aqueous solution, blood plasma, or precipitated blood plasma solution prior to the SERS analysis.

#### 6.2.8 Data Analysis

Data analysis was performed using RAMANMETRIX, PYTHON and ORIGIN Pro software. RAMANMETRIX [63] was used for spectral pre‐processing, PYTHON was used for peak area and peak intensity calculation using Simpson‘s method [64]. All spectra were finally plotted using Origin Pro software.

#### 6.2.9 Statistical Analysis

Spectral pre‐processing (wavenumber calibration and baseline correction) was carried out as described above. Briefly, all spectra were calibrated on the *x*‐axis using a standard acetaminophen spectrum and baseline‐corrected using the SNIP algorithm. For each experimental condition, SERS data were reported as mean ± standard deviation (SD), unless stated otherwise. The typical sample size was *n* = 3 independent batches × 3 substrates per batch, with 50 randomly acquired spectra per substrate; exact *n* values were specified in the figure legends. Calibration curves (Figures S7 and S12) were obtained by linear regression of integrated peak area versus analyte concentration. Limits of detection (LOD) and quantification (LOQ) were calculated from the regression parameters using standard criteria (LOD ≈ 3σ_blank/slope, LOQ ≈ 10σ_blank/slope, with σ_blank denoting the SD of the blank). Statistical evaluation was therefore based on descriptive statistics (mean, SD, RSD) and regression‐derived analytical performance metrics.

## Author Contributions

A.D. did methodology, conceptualization, experiment, data processing, and writing – original draft, review, and editing. J.D. did an experiment, editing. A.M. did the experiment and data processing. S.E.M. did a scientific discussion. V.S. did conceptualization, experiment, Data processing, funding acquisition, and writing – review. J.P. did supervision, funding acquisition, and writing – review. D.C.M. did supervision, conceptualization, funding acquisition, and writing – review. All authors have read and agreed to the published version of the manuscript.

## Conflicts of Interest

The authors declare no conflicts of interest.

## Supporting information




**Supporting File**: advs73837‐sup‐0001‐SuppMat.pdf.

## Data Availability

The data that support the findings of this study are available from the corresponding author upon reasonable request.
