# Supplementary material for: Sulfate‐Directed Silver Dendrites with Enhanced Stability for Ultrasensitive SERS‐Based Therapeutic Drug Monitoring
Source: Adv Sci (Weinh). 2026 Jan 18;13(17):e17092. doi: 10.1002/advs.202517092 (PMC13042930; doi:10.1002/advs.202517092)
Supplement: Supplementary file 1 — Supporting File: advs73837‐sup‐0001‐SuppMat.pdf. [file ADVS-13-e17092-s001.pdf]

Sulfate-Directed Silver Dendrites with Enhanced Stability for Ultrasensitive SERS-Based Therapeutic Drug Monitoring

Aradhana Dwivedi[a], Jan Dellith[a], Anna Makarova[c] Samir F. El-Mashtoly[a], , Juergen Popp[a,b], Vladimir Sivakov[a] and Dana Cialla-May[a,b,*]

[a] Leibniz Institute of Photonic Technology, Member of Leibniz Health Technologies, Member of the Leibniz Centre for Photonics in Infection Research (LPI), Albert-Einstein-Straße 9, 07745 Jena, Germany
[b] Institute of Physical Chemistry (IPC) and Abbe Center of Photonics (ACP), Friedrich Schiller University Jena, Member of the Leibniz Centre for Photonics in Infection Research (LPI), Helmholtzweg 4, 07743 Jena, Germany
[c] Helmholtz-Zentrum Berlin für Materialien und Energie, Hahn-Meitner-Platz 1, 14109 Berlin, Germany
*Corresponding author: dana.cialla-may@leibniz-ipht.de, dana.cialla-may@uni-jena.de

Table of contents:

Table S1: Comparison of different silver dendrite-based SERS substrates.
Table S2. Calculated analytical enhancement factor (AEF) values for 4-MBA.
Table S3: Detection (LOD) and quantification (LOQ) limits of our substrates for all drugs.
Figure S1: Schematic of proposed synthesis process comparison between silver nitrate and silver sulfate precursors.
Figure S2: Time resolved SEM images of silver sulfate dendrites formation at different growth stages.
Figure S3. Comparative SEM images of silver nanostructures synthesized via galvanic replacement using different silver salts under identical conditions.
Figure S4: Schematic illustration of growth mechanism for silver nitrate vs. silver sulfate precursors due to inherent concentration gradient of silver atoms.
Figure S5: SEM micrograph of silver nitrate substrate showing sparse nanostructures, EDX analysis showcasing elemental distribution of silicon and silver, corresponding SERS sensitivity of silver nitrate substrates with 4MBA.
Figure S6: Average SERS sensitivity of 3 batches with 4MBA on dendrite substrates.
Figure S7: Calibration curve of 4-MBA, for sensitivity analysis.
Figure S8. Raman spectrum of 4-MBA (10 mM) acquired on bare Si and representative SERS spectra of 4-MBA acquired on Ag dendrite substrates at 10^{-10} M - 10^{-13} M.
Figure S9: One on one comparison of AgDs and AgAs with lowest detected concentration of 4MBA, and the substrate backgrounds.
Figure S10. XPS S 2p spectra of sulfate-derived AgDs measured immediately after growth (AgD-fresh) and after one month of ambient storage (AgD-old).
Figure S11: Sample-to-sample variability of SERS intensity across three substrate replicas from three batches (fourth batch in main text), with standard deviations, corresponding RSD values from all four batches and their 3 replicas.
Figure S12. Representative SERS areamaps on s-AgDs. Heatmaps (top) and corresponding 3D spectral plots (bottom) are shown for two 10×10 grids (100 spectra each, panels a and b) and one 20×20 grid (400 spectra, panel c) acquired under identical acquisition conditions.

Figure S13: Comprehensive SERS spectra of the drugs in aqueous and blood plasma, along with respective calibration curves.

Table S1: Comparison of different silver dendrite-based SERS substrates.

Substrate	Test analyte	LOD	Synthesis	matrix	Real Target	Reference
Hydrogel	Nile blue A	10^{-10} M	Galvanic on Cu	Urine	Lung cancer cell (carbonyl biomarkers)	[1]
Insertable planer Al	R6G	10^{-9} M	Electrochemical + ALD	Apple	R6G	[2]
Ag Nano dendrites			Electrokinetic	Saliva	Cocaine, heroin, THC, oxycodone	[3]
Meta surfaces	R6G, CV	10^{-8} M	Photoreduction	Water	Perfluorooctanoic acid	[4]
Filter paper	Nitenpyram	10^{-9} M	Galvanic on Zn	Apple swab	Nitenpyram	[5]
ZnO coated	R6G	10^{-10} M	Electrochemical + ALD	Not available	Not available	[6]
TiO_2 nanotube	R6G	10^{-12} M	Electro-deposition	Water	Ibuprofen	[7]
**Sulfate assisted**	**4-MBA**	**4.9×10^{-13} M**	**Galvanic on Si**	**Blood plasma**	**6-Thioguanine, Erlotinib, Methotrexate, Doxorubicin, Moxifloxacin**	**This Work**

R6G – rhodamine 6G, CV – crystal Violet, 4-MBA – 4-mercapto benzoic acid, ALD – atomic layer deposition

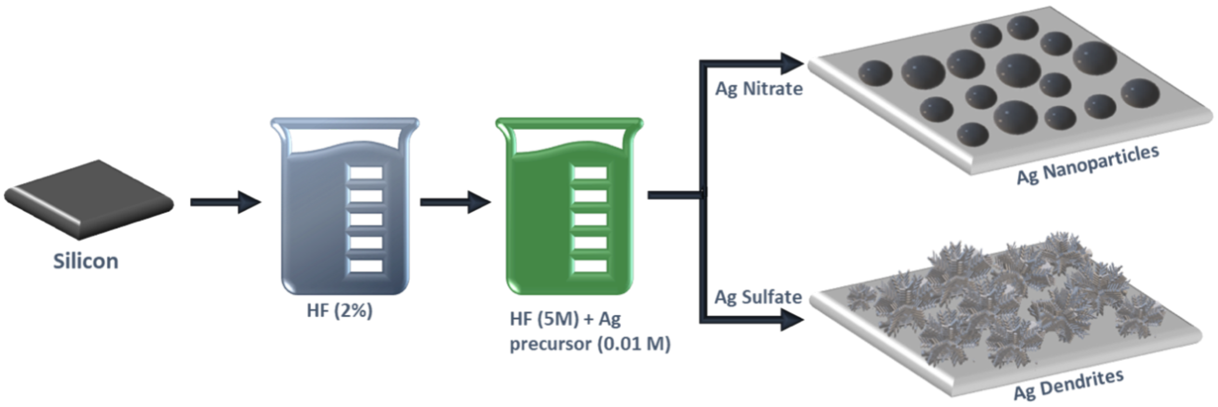

Figure S1: Schematic of proposed synthesis process comparison between silver nitrate and silver sulfate precursors.

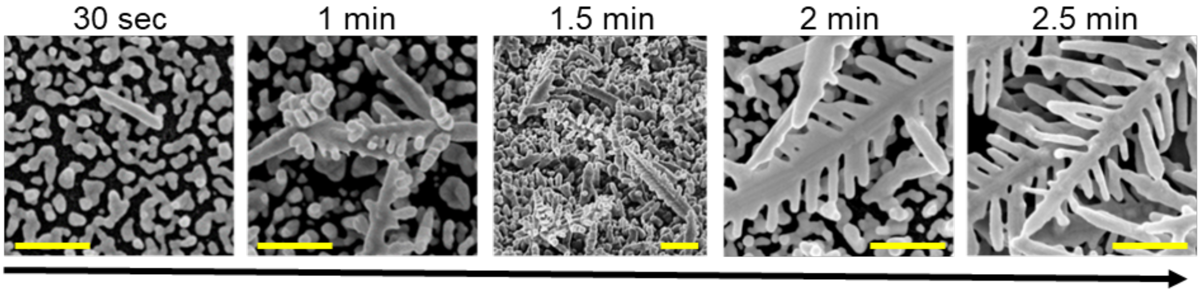

Figure S2: Time resolved SEM images of silver sulfate dendrites formation at different growth stages (all the scale bars are 500 nm).

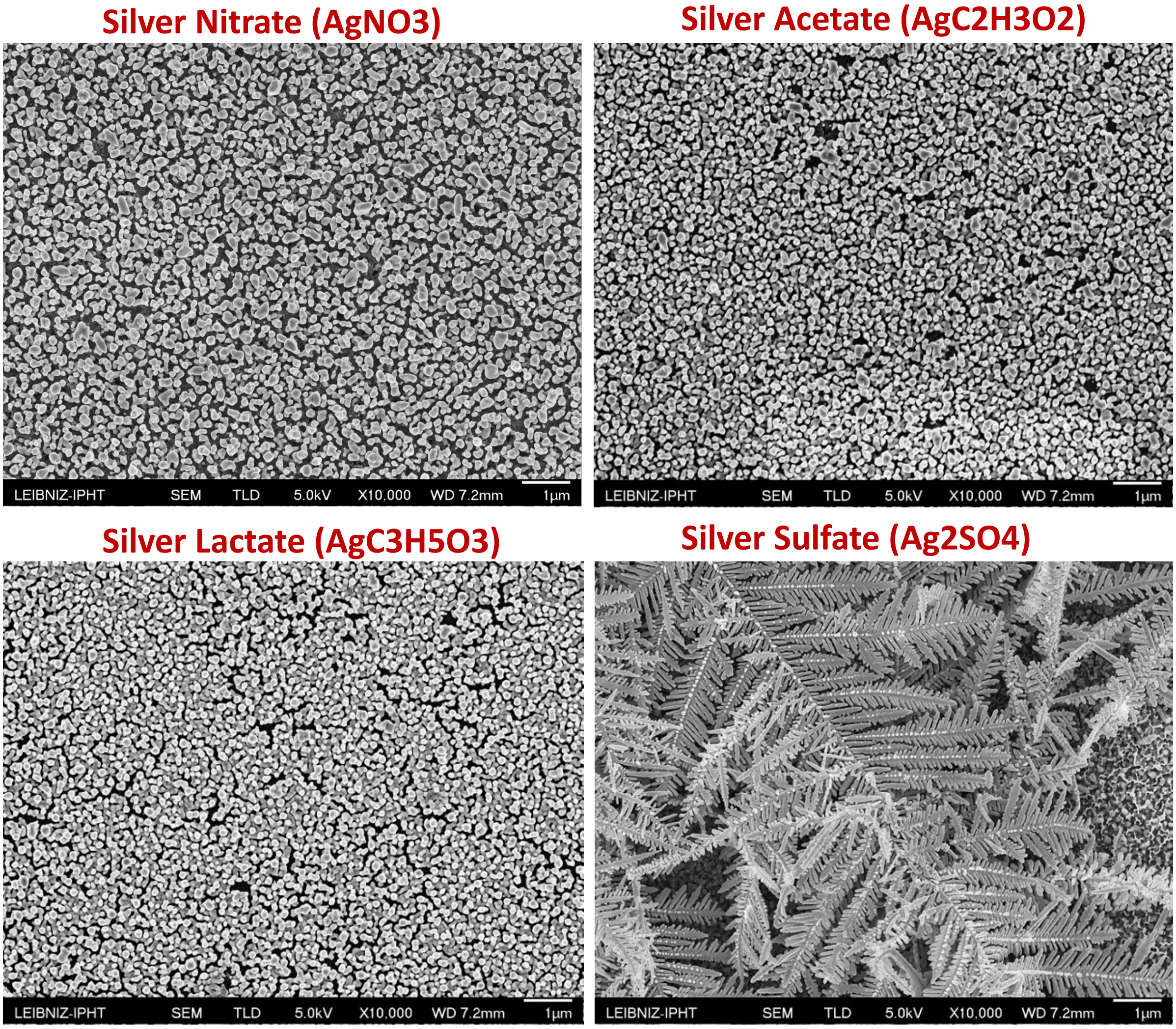

Figure S3. Comparative SEM images of silver nanostructures synthesized via galvanic replacement using different silver salts under identical conditions. Ag-nitrate, Ag-acetate, and Ag-lactate produced low-dimensional aggregates, while Ag-sulfate yielded multibranched dendrites in the same growth time.

To evaluate whether dendritic growth is specific to Ag-sulfate or can also be achieved with other silver salts, we compared Ag-nitrate, Ag-acetate, Ag-lactate, and Ag-sulfate under identical galvanic conditions (same molarity, etching time, and growth parameters). As shown in Figure S3, only Ag-sulfate produced highly branched, multijunction dendritic structures, while the other salts yielded compact, isotropic aggregates with limited structural complexity. This indicates that the dendritic morphology arises from a synergistic effect of the Ag-sulfate precursor, where the higher Ag^+ flux (2:1 stoichiometry) and the sulfate anion jointly promote rapid, anisotropic growth. These findings support the interpretation that sulfate is not merely a passive counterion, but actively promotes anisotropic dendrite formation.

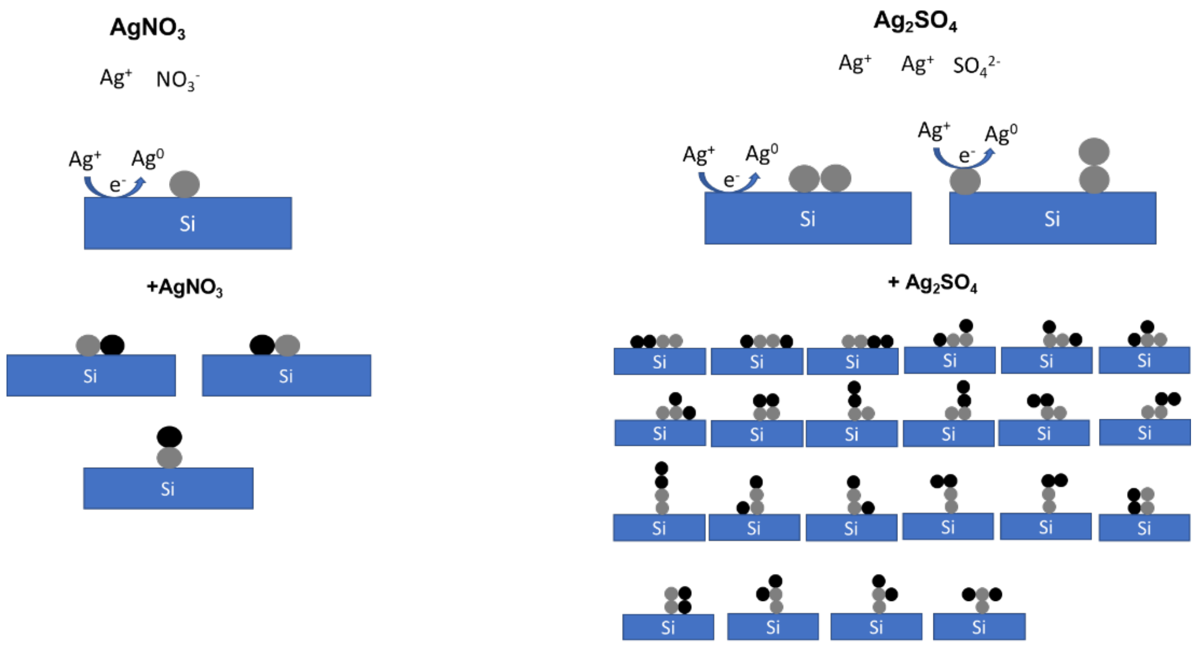

Figure S4: Schematic illustration of growth mechanism for silver nitrate vs. silver sulfate precursors due to inherent concentration gradient of silver atoms.

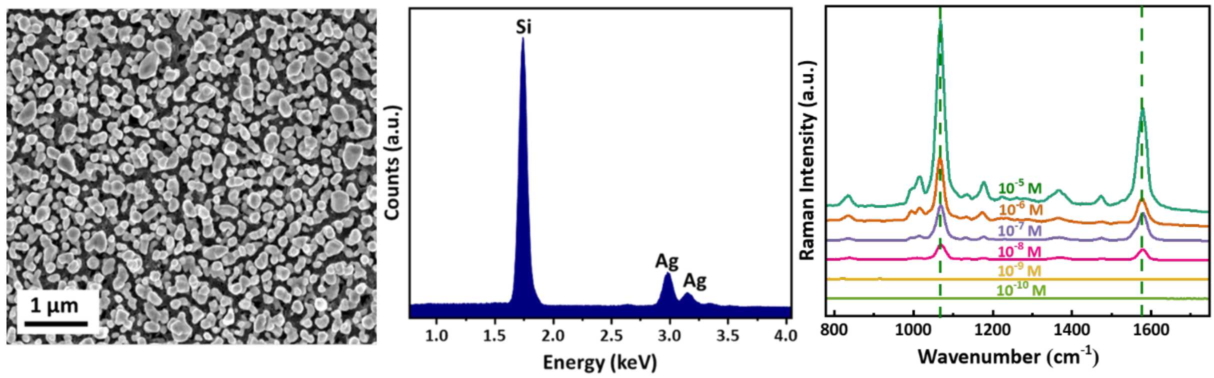

Figure S5: SEM micrograph of silver nitrate substrate showing sparse nanostructures, EDX spectrum showcasing the X-ray lines of Silicon (Ka-line at 1.74 keV) and silver (L-line around 3 keV), corresponding SERS sensitivity of silver nitrate substrates with 4MBA.

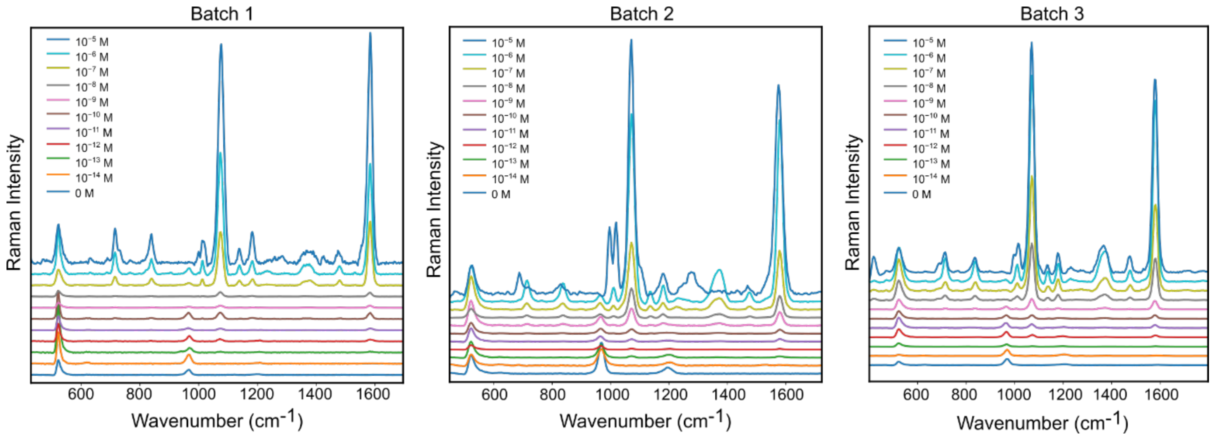

Figure S6: Mean SERS spectra for 4-MBA sensitivity measurements on s-AgDs for three independent batches (n = 3, 5 single spectra per concentration), corresponding to the batch-resolved data underlying the overall sensitivity curve shown in Figure 3a.

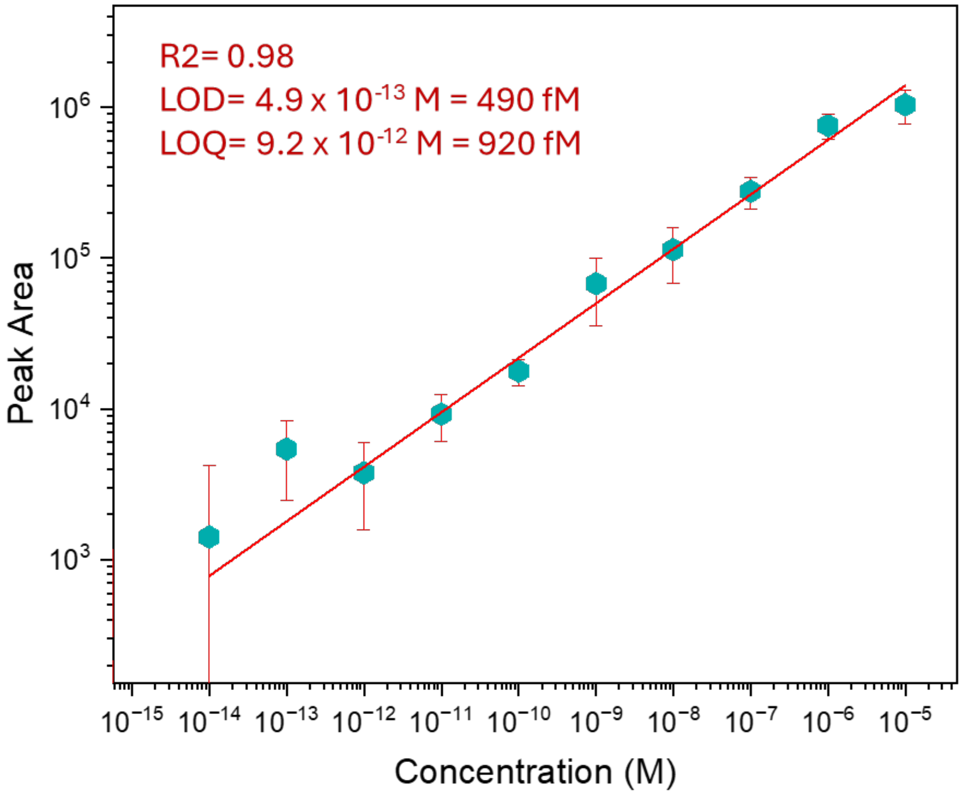

Figure S7: Calibration curve for 4-MBA on s-AgDs, obtained by plotting integrated peak area versus concentration for the spectra shown in Figure S6; data points represent mean values (n = 3 batches), and the solid red line indicates the linear regression used to derive LOD and LOQ.

Analytical enhancement factor (AEF):

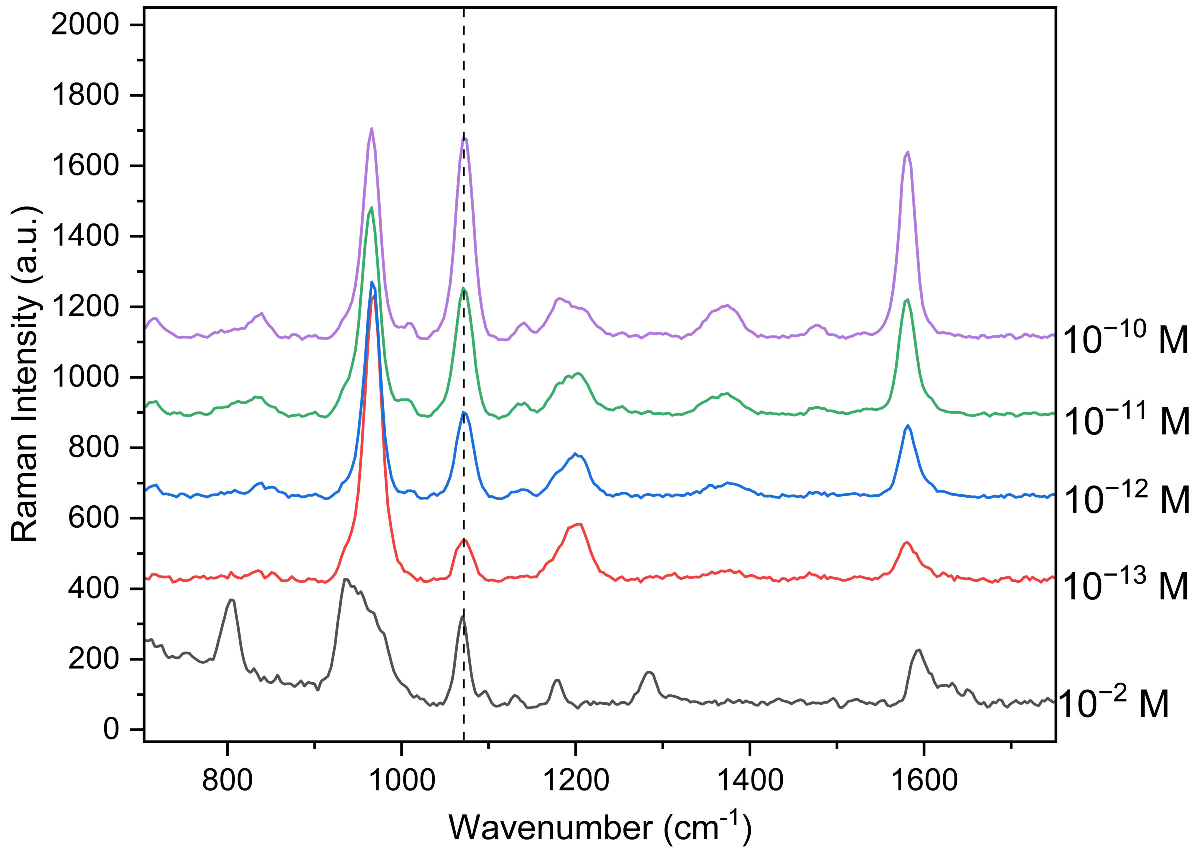

Figure S8. Raman spectrum of 4-MBA (10 mM) acquired on bare Si and representative SERS spectra of 4-MBA acquired on Ag dendrite substrates at 10^{-10} M - 10^{-13} M.

Table S2. Calculated analytical enhancement factor (AEF) values for 4-MBA.

MOLAR CONCENTRATION (M)	ENHANCEMENT FACTOR (AEF)
$\mathbf{10^{-10}}$	2.24×10^{8}
$\mathbf{10^{-11}}$	1.42×10^{9}
$\mathbf{10^{-12}}$	9.49×10^{9}
$\mathbf{10^{-13}}$	4.47×10^{10}

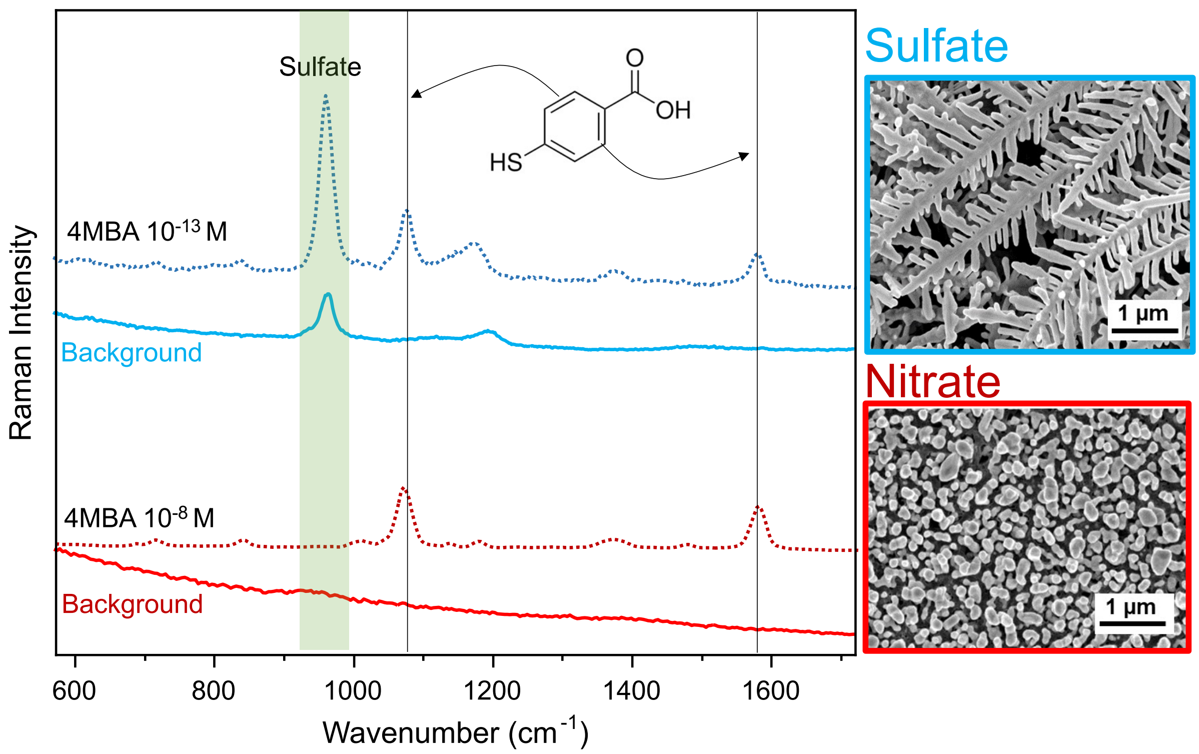

Figure S9: One on one comparison of AgDs and AgAs with lowest detected concentration of 4MBA and the substrate backgrounds.

The AgDs exhibit higher branch density and multiple junctions and nanogaps between branches, increasing hotspot probability. As shown in Fig. S9, when comparing sulfate-derived AgDs with nitrate-derived AgAs using 4-MBA as a model analyte, the lowest detectable concentration was ~10^{-13} M on AgDs versus ~10^{-8} M on AgAs. The reduced sensitivity of AgAs likely reflects their lower hotspot density and weaker field confinement. Importantly, a distinct sulfate-associated $v_1(SO_4^{2-})$ band is consistently observed at ~970 cm^{-1} in s-AgDs, but is absent in n-AgAs prepared under otherwise identical conditions and processed with the same washing, drying steps. This spectral feature corroborates the XPS evidence (S 2p doublets at ~162 and ~168 eV) and supports the interpretation that sulfate species remain associated with the AgD surface after growth and not due to the post-synthesis oxidation, consistent with their proposed role in passivation.

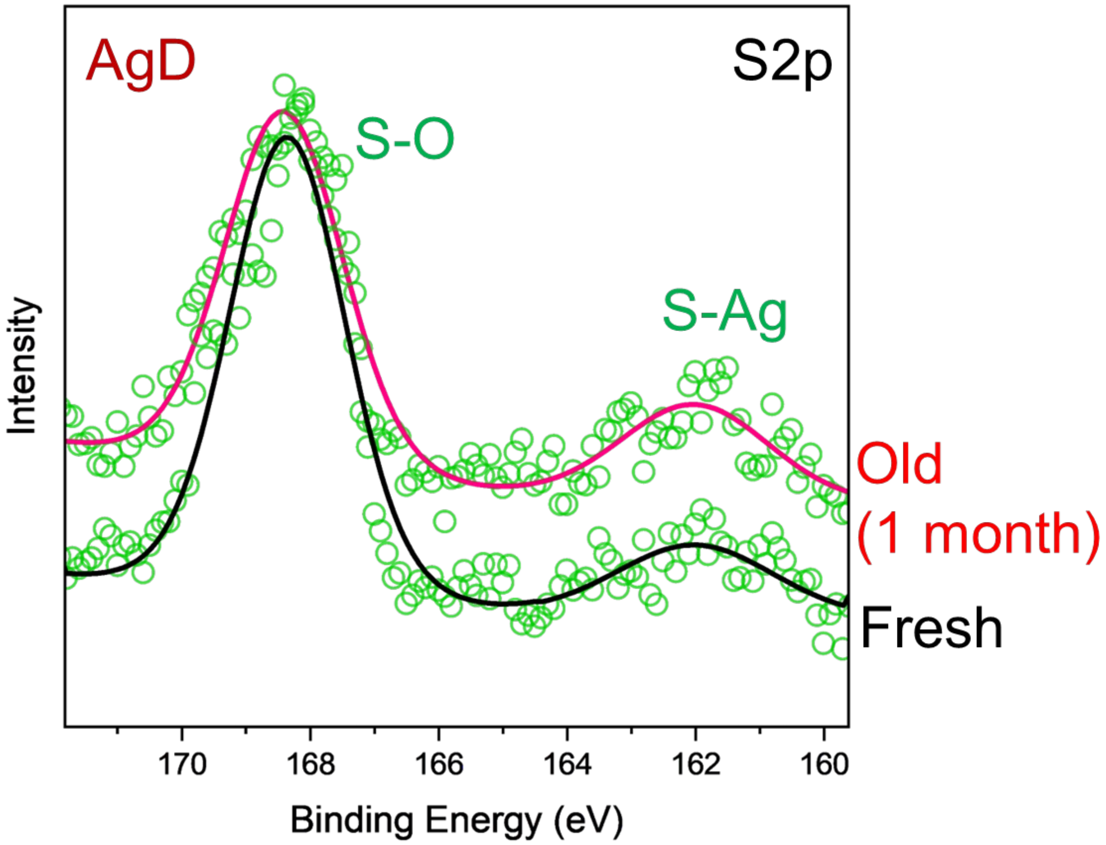

Figure S10. XPS S 2p spectra of sulfate-derived AgDs measured immediately after growth (AgD-fresh) and after one month of ambient storage (AgD-old). The characteristic sulfate-associated doublets remain clearly detectable in both samples.

To complement the functional SERS stability analysis, we performed synchrotron-based XPS on AgDs immediately after synthesis and after one month of ambient storage. As shown in Figure S10, the S 2p spectra exhibit the characteristic doublets at ~162 eV (S–Ag) and ~168 eV (S–O) in both cases, indicating that the sulfate species remain stably associated with the AgD surface even after prolonged ambient storage. This persistence supports the hypothesis that sulfate contributes not only to dendritic growth but also to the long-term stabilization of the surface against oxidation.

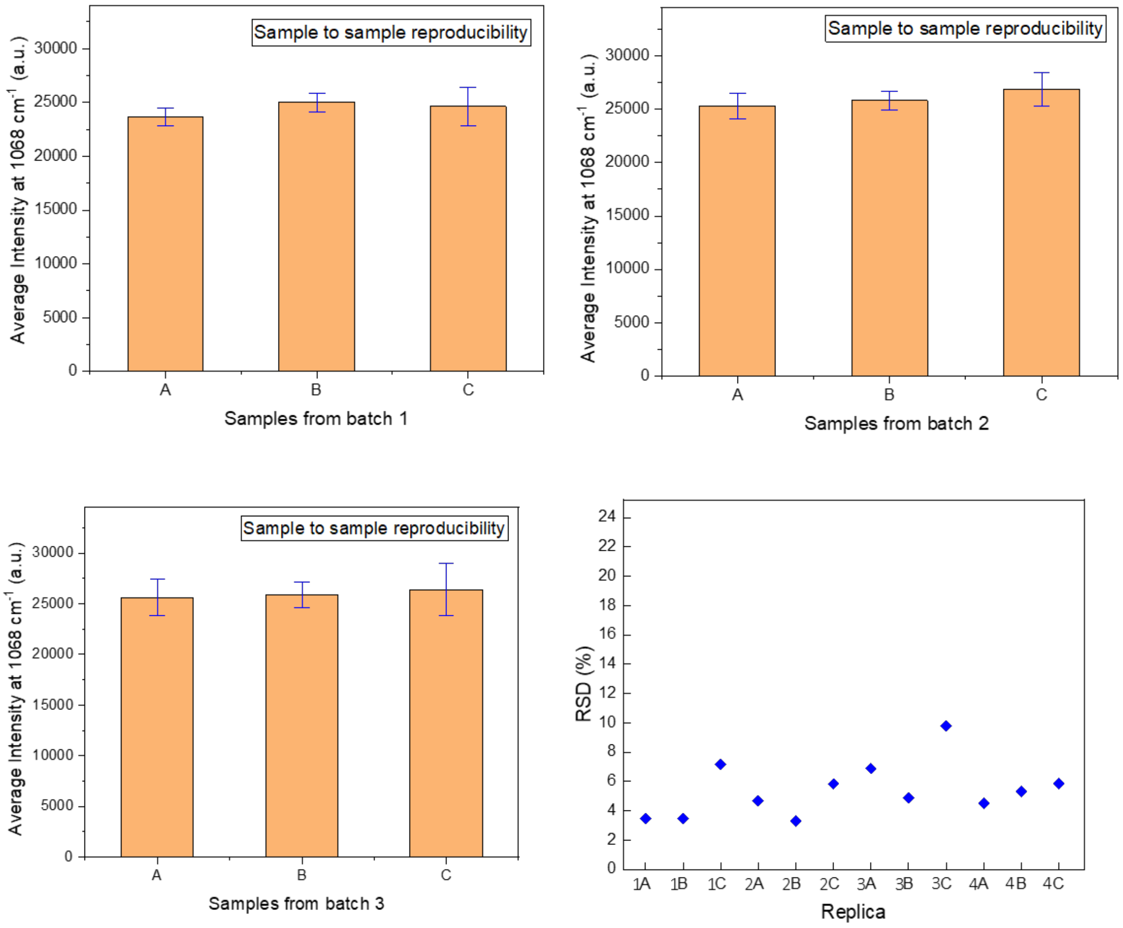

Figure S11: Sample-to-sample variability of SERS intensities at 1070 cm^{-1} for 4-MBA (10^{-5} M) across three substrate replicas (A–C) from three additional batches; data are shown as mean ± SD (50 spectra per substrate). The corresponding RSD values for all four batches (including batch 4 shown in the main text) and their three replicas are summarized to illustrate overall reproducibility.

Substrate Uniformity Assessed by SERS mapping:

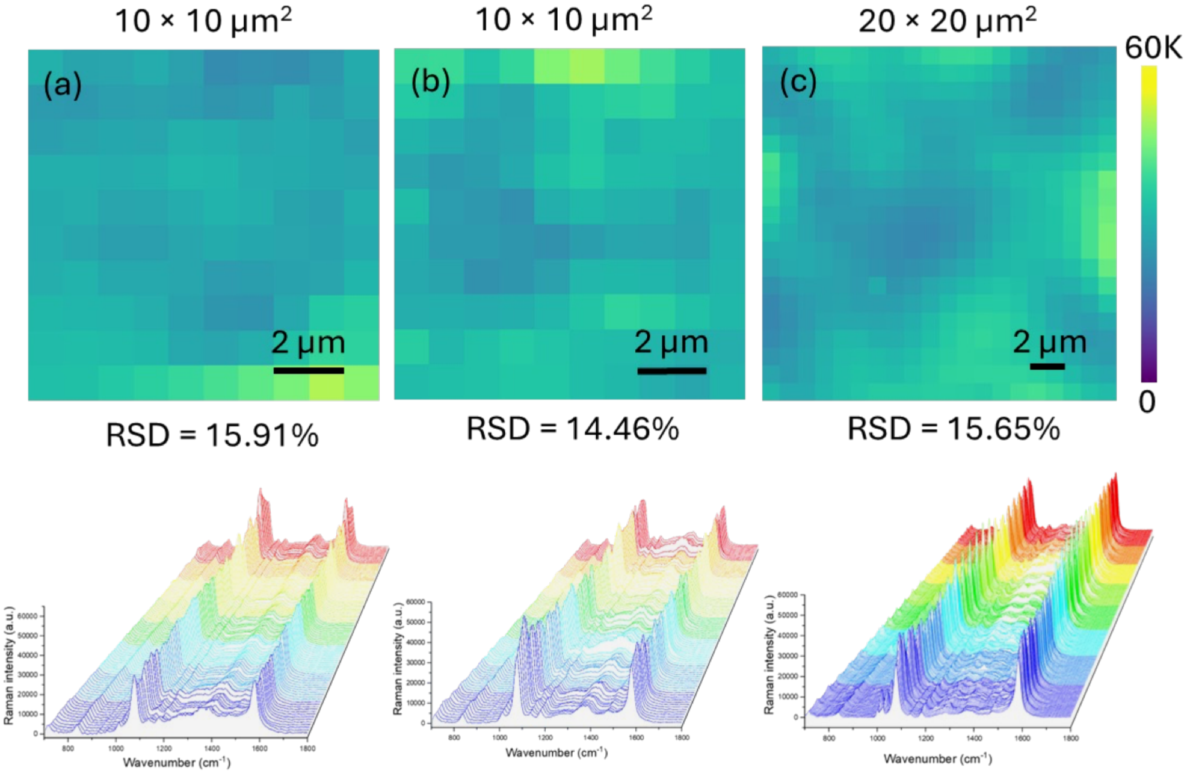

Figure S12. Representative SERS areamaps on s-AgDs. Heatmaps (top) and corresponding 3D spectral plots (bottom) are shown for two 10×10 grids (100 spectra each, panels a and b) and one 20×20 grid (400 spectra, panel c) acquired under identical acquisition conditions.

Figure S12 presents representative SERS areamaps acquired from sulfate-derived AgD substrates using 4-MBA. The heatmaps and 3D spectral plots correspond to two 10×10 μm² (100 spectra each) and one 20×20 μm² (400 spectra) measurement areas. As expected for 3D nanostructured surfaces, local signal intensity varies across the scan area due to differences in dendrite density, hotspot distribution, laser focus depth, and molecular adsorption. While local intensities vary due to topography and molecular distribution, replicate-averaged single-spot spectra (main text Fig. 3d,e) show consistent performance across batches.

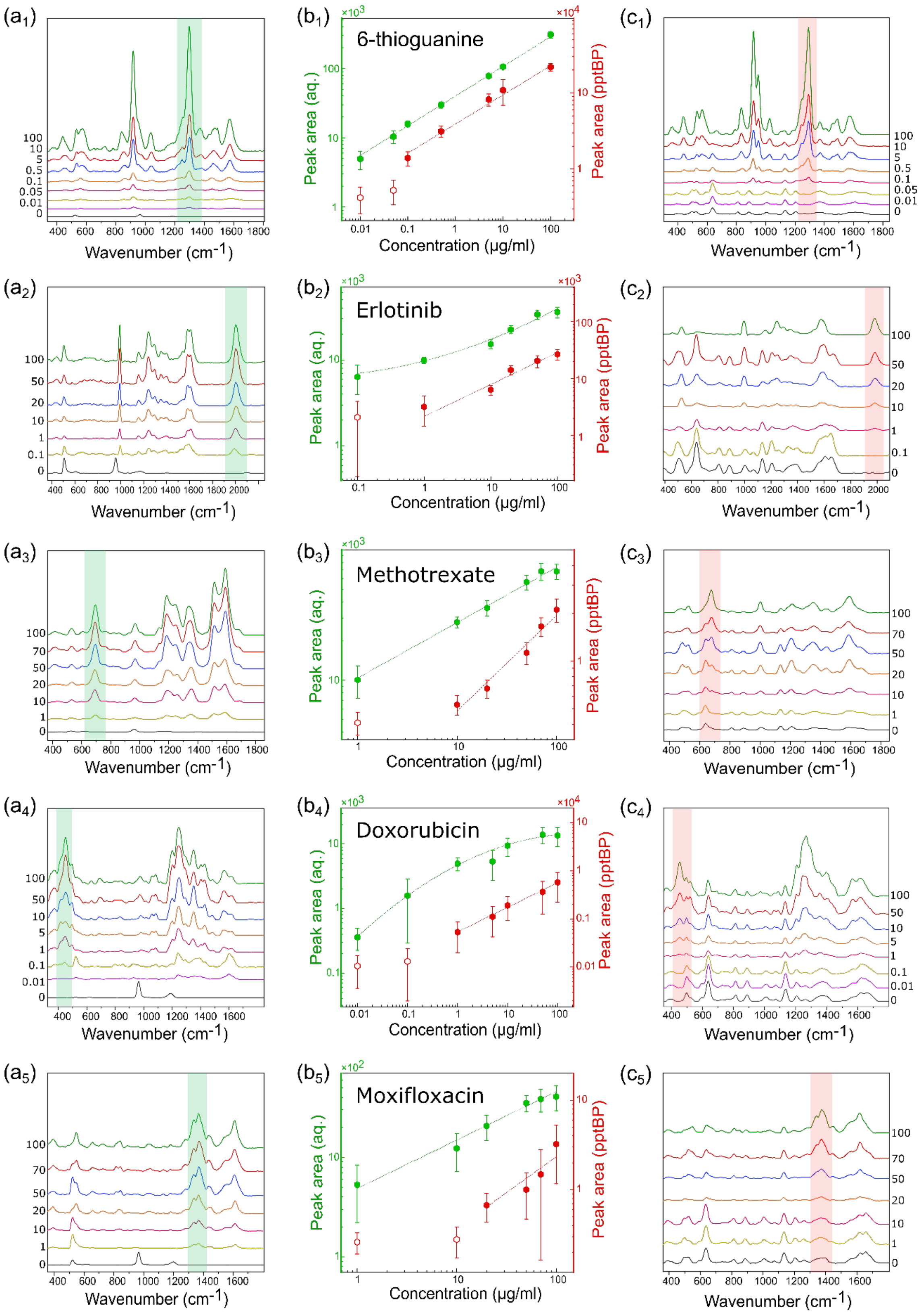

Figure S13: Average SERS spectra and calibration data for each drug obtained from three independent sample batches (n = 3). (a1–a5) Mean SERS spectra in aqueous solution over the respective concentration ranges. (b1–b5) Calibration curves for each drug, with green symbols/lines representing aqueous solution and red symbols/lines representing pretreated plasma (aq = aqueous solution, pptBP = precipitated blood

plasma). (c1–c5) Mean SERS spectra of the drugs spiked into and pretreated in blood plasma over the corresponding concentration ranges.

Blood plasma is a valuable matrix for therapeutic drug monitoring, but its complexity poses major challenges for SERS detection. Plasma contains thousands of molecules, including proteins, metabolites, salts, and nucleotides, that can adsorb to metallic surfaces or bind to the drug of interest, leading to hotspot fouling, reduced sensitivity, and strong background signals [8]. Proteins are particularly problematic, as they tend to form a protein corona at the SERS surface or sequester a significant fraction of the analyte, thereby blocking its direct interaction with hotspots. To mitigate these effects, several strategies have been reported, including analyte separation by chromatography or magnetic beads, deproteinization, and protein precipitation[8,9]. In our study we employed acetonitrile (ACN)-based protein precipitation of drug-spiked plasma, followed by sonication and centrifugation. ACN alters the solvation of proteins, inducing aggregation and precipitation, which can then be separated by centrifugation. This straightforward pretreatment reduces matrix complexity while retaining the analyte in solution.

Figure S13 (a_1-a_5) presents overlaid SERS spectra of each drug across the aqueous concentration series, enabling direct visual comparison of peak intensities. For semi-quantitative analysis, a prominent vibrational marker band was selected, and the log-log plots of peak intensity versus drug concentration are shown in Figure S13 (b_1-b_5). This log-log approach offers a convenient linearization of the data, facilitates the estimation of dynamic range and extrapolation toward lower detection thresholds. Corresponding SERS spectra for drugs in pretreated blood plasma at identical concentrations are shown in Figure S13 (c_1-c_5). The dendritic substrates provided a clear, drug-specific Raman signature at lower concentrations with improved sensitivity, despite the presence of additional plasma background. Comparing slopes and intercepts between aqueous (green curves) and plasma matrices (red curves) in Figure (b_1-b_5), allowed assessment of signal intensities, enabling refined determination of clinically relevant detection limit of drugs (Table S3). Solid and open symbols distinguish between detected and non-detected specific drug marker modes.

Table S3: Detection (LOD) and quantification (LOQ) limits of our substrates for all drugs.

Drug	Matrix	R^2	LOD (μg/mL)	LOQ (μg/mL)
6-Thioguanine	Aq.	0.99	0.000603	0.0059
	Plasma	0.99	0.024	0.145
Erlotinib	Aq.	0.96	0.000114	0.0014
	Plasma	0.91	5.4	30
Methotrexate	Aq.	0.99	0.00182	0.02
	Plasma	0.97	14.8	36.9
Doxorubicin	Aq.	0.94	0.021	0.146
	Plasma	0.98	0.33	2.9
Moxifloxacin	Aq.	0.96	0.080	0.52
	Plasma	0.94	19	45

References:

1. Chen, Z. *et al.* Hydrogel based flexible wearable sweat sensor for SERS-AI monitoring treatment effect of lung cancer. *Sens Actuators B Chem* **427**, 137155 (2025).
2. Wang, X. *et al.* Atomic layer deposition assisted fabrication of insertable silver dendrites-based SERS substrates with high adhesion. *Appl Surf Sci* **640**, 158466 (2023).
3. Dies, H., Raveendran, J., Escobedo, C. & Docoslis, A. Rapid identification and quantification of illicit drugs on nanodendritic surface-enhanced Raman scattering substrates. *Sensors and Actuators B* **257**, 382–388 (2018).
4. Wang, X. Y. *et al.* Silver dendrite metasurface SERS substrates prepared by photoreduction method for perfluorooctanoic acid detection. *Spectrochim Acta A Mol Biomol Spectrosc* **310**, 123932 (2024).
5. Wang, Q. *et al.* Superhydrophobic SERS substrates based on silver dendrite-decorated filter paper for trace detection of nitenpyram. *Anal Chim Acta* **1049**, 170–178 (2019).
6. Wang, X. *et al.* Simultaneously improved SERS sensitivity and thermal stability on Ag dendrites via surface protection by atomic layer deposition. *Appl Surf Sci* **611**, 155626 (2023).

7. Arenas-Hernandez, A. & Pal, U. Enhanced SERS performance of cavity-hosting silver dendrites grown over TiO2 nanotubes. *Appl Surf Sci* **679**, 161196 (2025).
8. Dwivedi, A. *et al.* SERS-Driven Ceftriaxone Detection in Blood Plasma: A Protein Precipitation Approach. https://doi.org/10.3390/chemosensors12100213 (2024) doi:10.3390/chemosensors12100213.
9. Liu, C. *et al.* Toward SERS-based therapeutic drug monitoring in clinical settings: Recent developments and trends. *TrAC Trends in Analytical Chemistry* **164**, 117094 (2023).
